# Elacestrant demonstrates strong anti-estrogenic activity in PDX models of estrogen-receptor positive endocrine-resistant and fulvestrant-resistant breast cancer

**DOI:** 10.1038/s41523-022-00483-1

**Published:** 2022-11-29

**Authors:** Sunil Pancholi, Nikiana Simigdala, Ricardo Ribas, Eugene Schuster, Mariana Ferreira Leal, Joanna Nikitorowicz-Buniak, Camilla Rega, Teeru Bihani, Hitisha Patel, Stephen R. Johnston, Mitch Dowsett, Lesley-Ann Martin

**Affiliations:** 1 Breast Cancer Now Toby Robins Research Centre, The Institute of Cancer Research, London, SW7 3RP UK; 2 Radius Health, Waltham, Massachusetts, MA 02451 USA; 3 Breast Unit, Royal Marsden Hospital, London, SW3 6JJ UK; 4 Ralph Lauren Centre for Breast Cancer Research, Royal Marsden Hospital, London, SW3 6JJ UK

**Keywords:** Breast cancer, Breast cancer, Pharmaceutics

## Abstract

The selective oestrogen receptor (ER) degrader (SERD), fulvestrant, is limited in its use for the treatment of breast cancer (BC) by its poor oral bioavailability. Comparison of the orally bioavailable investigational SERD elacestrant, versus fulvestrant, demonstrates both drugs impact tumour growth of ER+ patient-derived xenograft models harbouring several *ESR1* mutations but that elacestrant is active after acquired resistance to fulvestrant. In cell line models of endocrine sensitive and resistant breast cancer both drugs impact the ER-cistrome, ER-interactome and transcription of oestrogen-regulated genes similarly, confirming the anti-oestrogenic activity of elacestrant. The addition of elacestrant to CDK4/6 inhibitors enhances the antiproliferative effect compared to monotherapy. Furthermore, elacestrant inhibits the growth of palbociclib-resistant cells. Lastly, resistance to elacestrant involves Type-I and Type-II receptor tyrosine kinases which are amenable to therapeutic targeting. Our data support the wider clinical testing of elacestrant.

## Introduction

Over 80% of breast cancer (BC) patients have oestrogen receptor-positive (ER+) tumours, which proliferate in response to oestrogen (E)^1^. E-bound-ER binds to E-response elements (EREs) within the promoters of genes, controlling proliferation and survival. Clinically, patients with ER + BC are treated with endocrine therapies that either block the production of E using aromatase inhibitors (AI), or that antagonise E activity by competitively binding the ER, such as tamoxifen, a selective ER modulator (SERM)^2^. Despite the efficacy of these agents, a large proportion of patients’ relapse with either de novo or acquired resistant disease, the majority of them continuing to express a functional ER (reviewed by^3^).

Fulvestrant is a pure anti-E drug approved for clinical use. It acts by blocking the transcriptional activity of ER, by impeding AF1 and AF2 thereby reducing the half-life of ER and is termed a selective ER downregulator/degrader (SERD) and is as effective as anastrozole^4^ in the treatment of ER+ advanced BC. ER downregulation has been shown to be a dose-dependent process and evidence correlates greater ER downregulation with superior efficacy^5^. However, fulvestrant is currently limited by its poor oral bioavailability and only reaches a steady state after 3–6 months of monthly intramuscular injections. The rationale for an oral SERD with improved pharmaceutical properties is to achieve higher steady-state levels, providing a better partner for combination therapy with other agents (e.g. CDK4/6 or PI3K pathway inhibitors)^6,7^ and allowing long-term treatment in the adjuvant setting. In the search for alternative therapies, it is key that oral compounds have a similar profile of pure anti-oestrogenic activity, with the ability to downregulate ER and suppress ER-regulated gene transcription^8^. Furthermore, recent studies have highlighted the importance of *ESR1* mutations in patients with metastatic ER + BC, which have been shown to have decreased sensitivity to endocrine therapy, thereby requiring higher doses of the relevant drugs^9–12^. To date, a number of oral SERDs have been in preclinical and clinical development^13–15^.

In the following study, we explored the efficacy of the oral SERD, elacestrant^16^, in vitro and in PDX models of endocrine-sensitive and endocrine-resistant ER + BC expressing various naturally occurring *ESR1* mutations. Elacestrant is currently under investigation in phase III clinical trial (EMERALD) (NCT03778931) in patients with ER+/HER2-negative advanced BC^17^, including patients whose tumours harbour *ESR1* mutations.

Our findings highlight the ability of elacestrant to downregulate ER target genes similar to fulvestrant but with greater activity in vivo. Furthermore, we provide evidence that supports the combination of elacestrant with other targeted therapies such as CDK4/6 and mTORC1/PI3K inhibitors for use in endocrine or palbociclib-resistant BC. Finally, we established a model of resistance to elacestrant that can be targeted with inhibitors against growth factor receptors.

## Results

### Comparison of the antiproliferative effect of elacestrant versus fulvestrant in isogenic models of endocrine sensitive and resistant BC

**Fig. 1:**
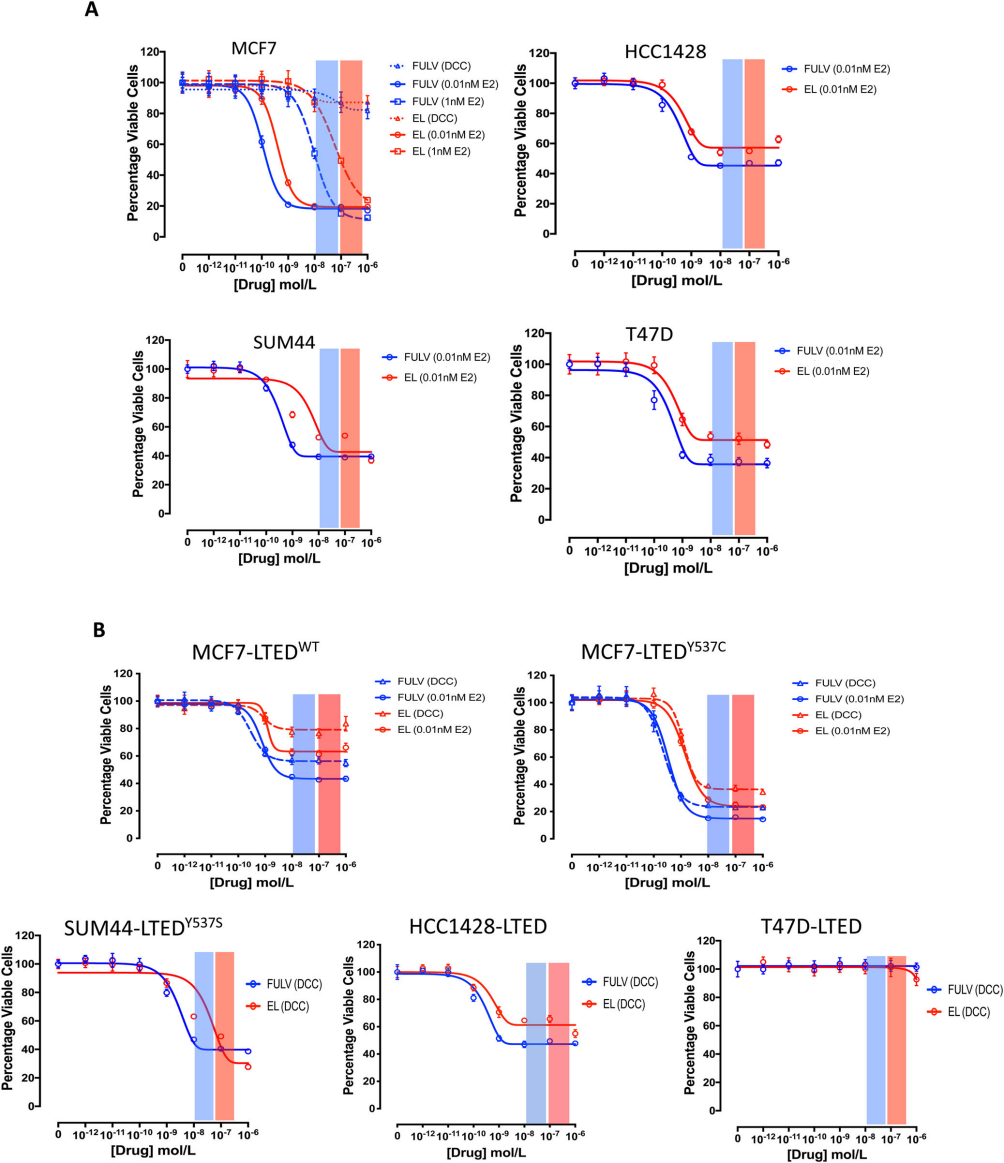
Effect of elacestrant versus fulvestrant on cell proliferation. Effect of escalating concentrations of elacestrant (EL) or fulvestrant (FULV) in models of **A** endocrine sensitive and **B** resistant cell lines. MCF7 cells were treated in DCC medium in the absence of 17β-estradiol (E2) (modelling E-deprivation on an AI) or in the presence of 0.01 and 1 nM E2. All other parental cell lines were tested in the presence of 0.01 nM E2. MCF7-LTED cell lines were tested in a DCC medium in the absence or presence of 0.01 nM E2. All other LTED models were tested in DCC alone. Cell viability was analysed using a CellTiter-Glo assay. Data were expressed as a percentage of viable cells in relation to vehicle control. Error bars represent means ± SEM. Red and blue shaded bars represent the clinically achievable doses for elacestrant and fulvestrant, respectively (*n* = 3 biological experiments with six technical replicates per treatment).

To test the antiproliferative effect of elacestrant versus fulvestrant in models of ER + BC, we used a panel of isogenic cell lines modelling sensitivity or resistance to E-deprivation (LTED) for which the *PIK3CA, PTEN* and *ESR1* mutation status was previously established^12,18^ (Supplementary Table 1). We further assessed the antiproliferative effect of both drugs in a CRISPR-Cas9-engineered MCF7-LTED, where a Y537C mutation in *ESR1* was reverted to wild-type (wt) (MCF7-LTED^∆537C^)^12^. In the presence of 0.01 nM exogenous estradiol (E2), all parental cell lines showed a concentration-dependent decrease in cell viability in response to both drugs. On average, the IC_50_ for elacestrant was 10-fold higher than for fulvestrant; however, the IC_50_ values for both agents were within the clinically achievable concentration ranges of 10–30 nM for fulvestrant and 100–300 nM for elacestrant (Fig. 1A)^5,16,19^. In addition, MCF7 were also tested in the presence of 1 nM exogenous E2 and demonstrated a similar response, with IC_50_ values within the clinically achievable range (Fig. 1A). In order to assess the added benefit of combining elacestrant with E-deprivation, modelling the addition of an AI, MCF7 cells were cultured in the absence of exogenous E2 and with escalating concentrations of both SERDs. As expected, E-deprivation alone reduced cell viability significantly and the addition of either fulvestrant or elacestrant, caused minimal further reduction (Fig. 1A and Supplementary Fig. 1A).

We next explored the effect of fulvestrant or elacestrant in cell lines modelling acquired resistance to LTED. In this setting, we compared three isogenic MCF7-LTED models, either homozygote for *ESR1*^wt^, heterozygote *ESR1*^Y537C^ or *ESR1*^∆537C^ (CRISPR-Cas9 revertant) (Fig. 1B and Supplementary Fig. 1B). Escalating concentrations of both fulvestrant and elacestrant, in the presence or absence of E2, caused a concentration-dependent decrease in cell viability in MCF7-LTED^wt^; however, the response was less pronounced compared to MCF7-LTED^Y537C^. As expected, the addition of E2 appeared to decrease the surviving fraction in both MCF7-LTED^wt^ and MCF7-LTED^Y537C^ (Fig. 1B). In order to confirm specificity, MCF7-LTED^∆Y537^ were treated with fulvestrant or elacestrant in the absence of E2 and showed little impact on cell viability compared to the effects of the SERDs in the presence of E2, recapitulating the profiles seen for MCF7. This confirmed firstly that the *ESR1*^Y537C^ mutation governs the resistant phenotype and secondly that ER is targeted by elacestrant (Supplementary Fig. 1B). Furthermore, the antiproliferative effect of elacestrant was confirmed in 3D spheroid models of MCF7 and both MCF7-LTED cell lines (Supplementary Fig. S1C).

We next assessed the efficacy of fulvestrant and elacestrant in an extended panel of LTED models. Elacestrant and fulvestrant effectively suppressed the proliferation of SUM44-LTED^Y537S^, while HCC1428-LTED, which overexpress *ESR1*^*wt*^, showed a similar sensitivity profile to that seen for MCF7-LTED^wt^. Lastly, T47D-LTED, which loses expression of *ESR1* at the point of resistance, showed no sensitivity to either SERD, as expected (Fig. 1B).

Next, we addressed the potential activity of both drugs after the acquisition of resistance to tamoxifen or fulvestrant (Supplementary Fig. 1D and S1E). MCF7 cells with acquired resistance to tamoxifen (MCF7-TAMR) showed sensitivity to escalating concentrations of both SERDs (Supplementary Fig. 1D). Contrastingly, MCF7-LTED-ICIR cells, which reduce *ESR1* expression as part of the resistance mechanism showed no sensitivity to elacestrant (Supplementary Fig. 1E). Taken together these data confirm the ability of elacestrant to specifically antagonise ER-function in both endocrine-sensitive and endocrine-resistant models where ER is expressed and remains the mitogenic driver.

### The effects of elacestrant and fulvestrant on ER protein inhibition and degradation

**Fig. 2:**
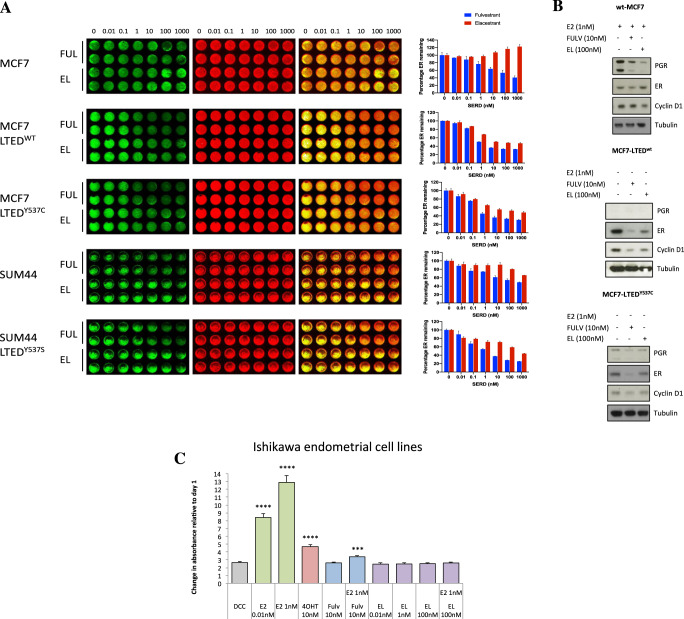
Effect of elacestrant on ER degradation. **A** InCell Western blot for MCF7, MCF7-LTED^wt^, MCF7-LTED^Y537C^, SUM44 and SUM44-LTED^Y537S^ following a concentration range of fulvestrant (FULV) or elacestrant (EL). ER (green), nuclear marker- IRDye 800CW (red) and overlay (yellow). Bar graphs on the right panels represent the percentage of ER remaining after treatments (*n* = 3 biological replicates with two technical replicates per treatment). **B** Immunoblotting of MCF7, MCF7-LTED^wt^ and MCF7-LTED^Y537C^ showing protein abundance of PGR, ER and Cyclin D1 after treatment with fulvestrant or elacestrant (data shown is representative of *n* = 2 experiments). **C** Alkaline phosphatase assay highlighting changes in absorbance after treatment with estradiol (E2), 4OHT, fulvestrant or increasing concentrations of elacestrant relative to day 1 in Ishikawa endometrial cell line. Bars represent the mean ± SEM of *n* = 3 independent experiments with *n* = 6 technical replicates. ****p < 0.0001, ***p < 0.001 (one way ANOVA, with Dunnett’s multiple comparisons test).

SERDs such as fulvestrant act by binding to ER, impairing dimerisation and accelerating proteasomal degradation. In order to assess the mode of action of elacestrant in comparison to fulvestrant, an InCell western assay was carried out to examine ER protein levels following treatment with both drugs. In MCF7 cells, fulvestrant reduced ER levels in a dose-dependent manner, while elacestrant led to a measurable increase in ER levels, a finding that may be related to increased stability of the complex (Fig. 2A). Across the remaining cell lines tested, fulvestrant demonstrated a greater ability to reduce levels of ER at lower doses than elacestrant after 18 h treatment. An examination of select markers revealed a reduction in protein levels of PGR and cyclin D1 upon treatment with either drug, suggesting again that both drugs were effective at blocking ER-downstream activity (Fig. 2B). However, ER levels matched those observed in the InCell assay with slight decreases in both MCF7-LTED derivatives but a noticeable increase in MCF7 cells, suggesting that elacestrant’s SERD properties may differ from those of fulvestrant, depending on the context.

To determine whether elacestrant had SERM-like agonist activity similar to tamoxifen in other endocrine tissues, the Ishikawa endometrial cell line was used in which alkaline phosphatase activity is regulated by ER. As expected, the addition of E2 led to substantially increased expression of alkaline phosphatase (Fig. 2C). Tamoxifen caused a small but significant increase in expression, however, both fulvestrant and elacestrant caused no increase in alkaline phosphatase levels, indicating a lack of agonist activity in the endometrial model. In a competition assay, fulvestrant was better at blocking the agonist activity of exogenous E2 (0.01 or 1 nM) than elacestrant (Supplementary Fig. 2). These data suggest that elacestrant is a pure anti-E.

### Comparison of the effect of elacestrant versus fulvestrant on ER-function

**Fig. 3:**
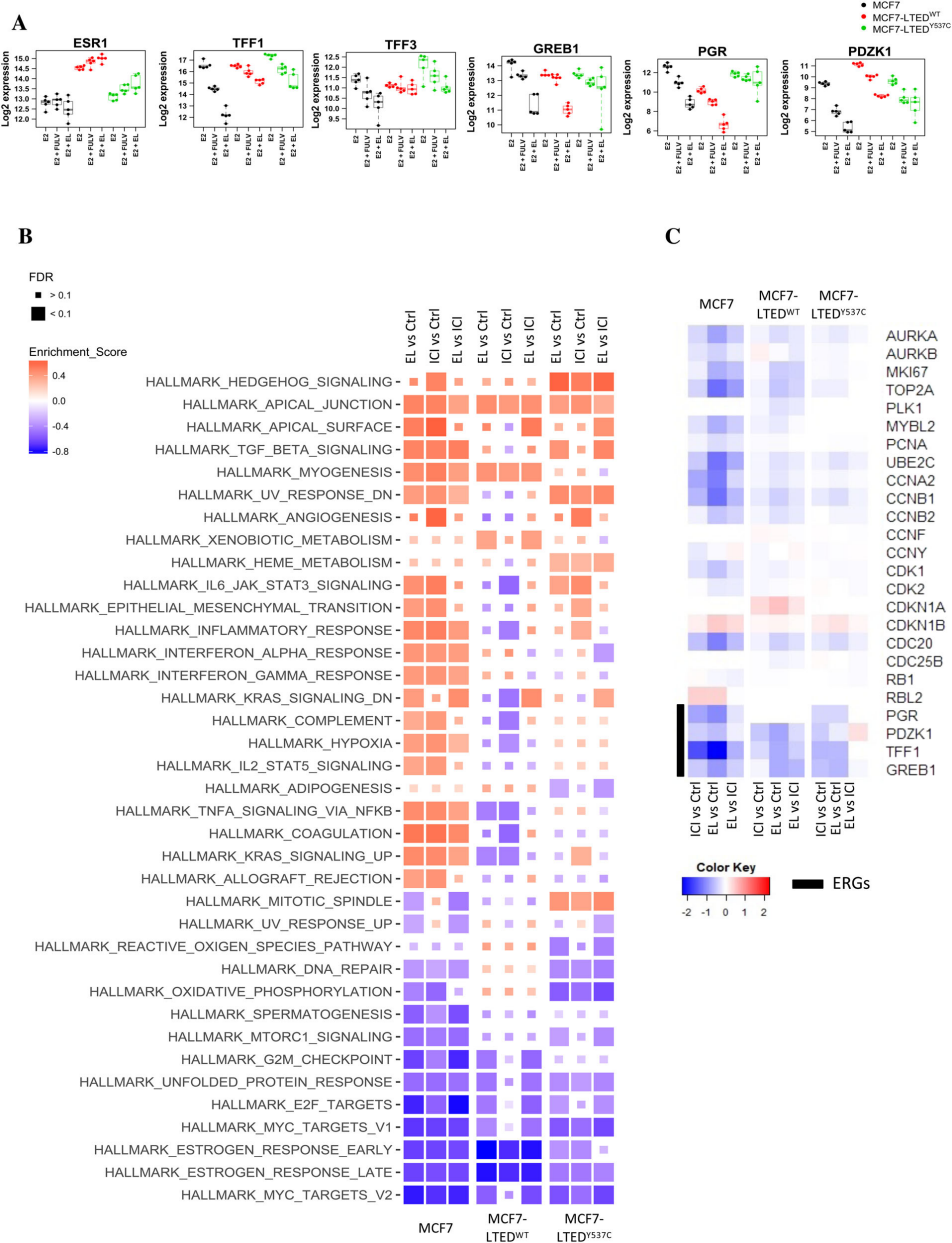
Effect of elacestrant versus fulvestrant on ER-mediated transcription and translation. **A** Log2 expression of E-regulated genes together with *ESR1* in MCF7 and both MCF7-LTED cell lines after treatment with 10 nM fulvestrant or 100 nM elacestrant. All treatments were performed in the presence of 1 nM E2. **B** Heatmap displaying changes in hallmark pathways following treatment with 10 nM fulvestrant (FULV) or 100 nM elacestrant (EL) in MCF7, MCF7-LTED^wt^ and MCF7-LTED^Y537C^. **C** Effect of 100 nM elacestrant and 10 nM fulvestrant upon protein abundance of cell cycle markers and E-regulated proteins in MCF7, MCF7-LTED^wt^ and MCF7-LTED^Y537C^. (Data from *n* = 5 biological experiments, per treatment per cell line).

In order to compare the effect of elacestrant versus fulvestrant on global gene expression and protein abundance, MCF7 and both MCF7-LTED isogenic lines were treated with each SERD in the presence of E2 in order to model the clinical scenario in which E2 levels rise as a result of a cessation of AI therapy at relapse. Elacestrant was more effective than fulvestrant at decreasing the expression of E-target genes: *TFF1*, *TFF3*, *PGR*, *PDZK1* and *GREB1* for all cell lines (Fig. 3A). Assessment of *ESR1* showed that elacestrant increased mRNA expression to a greater extent than fulvestrant in both MCF7-LTED models. Noteworthy, neither of the drugs significantly affected the mRNA expression of *ESR1* in the parental MCF7 (Fig. 3A).

Pathway analysis showed both elacestrant and fulvestrant caused a similar impact on signalling pathways in all cell lines (Fig. 3B), however, in MCF7-LTED^wt^, elacestrant caused subtle changes in pathways involved with proliferation, such as E2F and G2/M checkpoint as well as MYC-targets and KRAS, while fulvestrant enhanced pathways were involved in inflammation, such as inflammatory response, IL2/STAT5, IL6/JAK/STAT3, complement and hypoxia. Assessment of MCF7-LTED^Y537C^ showed that elacestrant also reduced proliferation pathways (E2F targets) as well as mTORC1 signalling (Fig. 3B). Global proteomic analysis further confirmed the effect of both SERDs on the inhibition of proliferation and E-response associated pathways in all three cell lines (Fig. 3C; Supplementary Fig. 3 and Supplementary File S1). Further analysis showed a lower abundance of ubiquitin and E3-ligases such as UBE2C, FXBO38 and FXBO33 in elacestrant-treated cell lines compared to fulvestrant. This may be linked to the higher ER protein abundance in elacestrant versus fulvestrant-treated cells.

Taken together, these data demonstrate that both elacestrant and fulvestrant target E-mediated transcription and cell proliferation.

### Comparison of the effect of elacestrant versus fulvestrant on the ER-cistrome

**Fig. 4:**
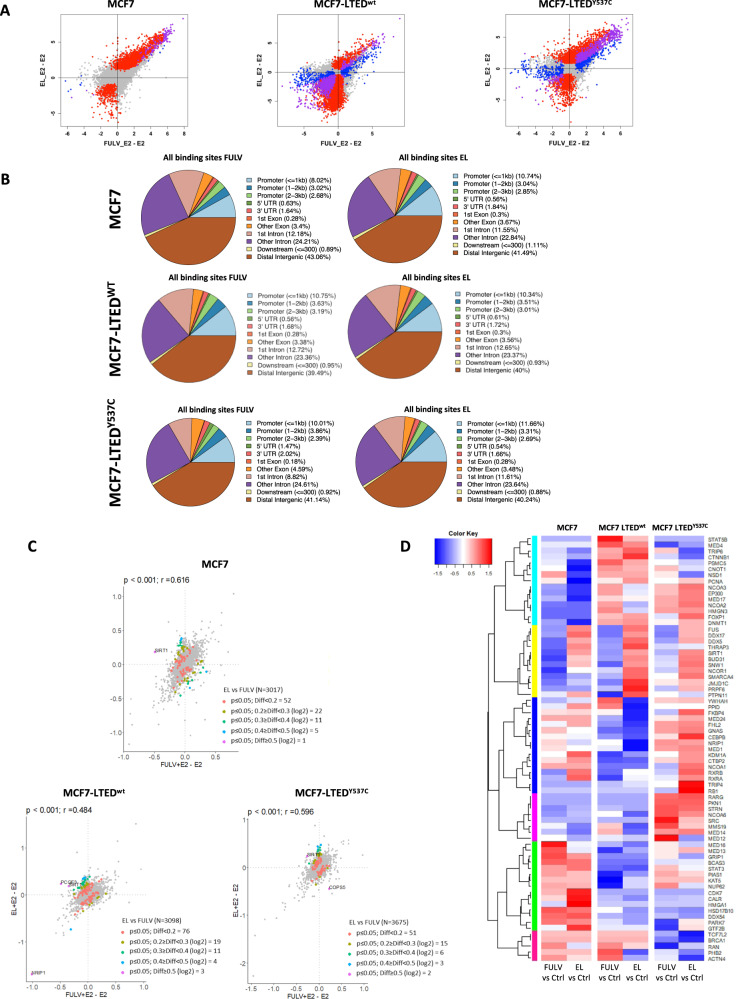
Effect of fulvestrant and elacestrant on the ER-binding in MCF7, MCF7-LTED^wt^ and MCF7-LTED^Y537C^. **A** ChIP-seq revealing similar effects of elacestrant (EL) and fulvestrant (FULV) on ER binding. Significantly differential ER binding (FDR < 0.05) as determined by DiffBind between EL + E2 and E2-only (red), between FULV + E2 and E2-only (blue) and overlap of significant binding sites (purple). **B** Genomic distribution of ER-binding sites. **C** RIME proteomics analysis revealing a similar effect of elacestrant (EL) and fulvestrant (FULV) on the ER-interactome. Different colour dots correspond to Log2 differences. **D** Heatmap showing GO hormone-binding proteins that interacted in cells treated with fulvestrant or elacestrant in relation to the control (E2). *p* value based on Student’s *t*-test or Pearson correlation is shown; r Pearson’s correlation score (Data from *n* = 2 biological replicates per treatment per cell line).

To further characterise the activity of elacestrant and fulvestrant on the genome-wide binding of ER, we performed ChIP-seq and Rapid immunoprecipitation, mass spectrometry of endogenous proteins (RIME) for ER (Fig. 4). ChIP-seq showed there was a highly significant correlation (*p* values < 10^−16^) between ER binding fold changes induced by the addition of elacestrant or fulvestrant in the three cell lines tested (Pearson correlations with *p* values <10^−16^; 0.78 MCF7, 0.47 MCF7-LTED^wt^ and 0.60 MCF7-LTED^Y537C^) especially in regions where there was increased ER binding after treatment versus E2 (Fig. 4A). There were 21,506 common ER binding sites in the three cell lines tested and only 281 binding sites with >2-fold difference between elacestrant or fulvestrant across the three cell lines (81 with greater suppression of ER binding after addition of elacastrant compared to the addition of fulvestrant and 200 with great suppression after addition of fulvestrant). However, assessment of RNA-seq data showed none of the genes that were mapped nearest to these sites showed a consistent up or downregulation at both the gene (FDR <0.05 in three cell lines) and protein (>25% change in expression levels) level.

The genes nearest the 200 ER binding sites with lower ER binding after addition of fulvestrant were not enriched for lower expression, as 35 had a higher expression with the addition of fulvestrant across the three cell lines (FDR <0.05) and 39 genes had lower expression. Similarly, the genes nearest the 81 ER binding sites with lower ER binding after the addition of elacestrant had an equal number of genes with higher and lower expression (*n* = 13).

Moreover, both drugs showed similar patterns of genomic distribution (Fig. 4B). The intersection of differentially expressed genes (identified by RNA-seq) and of genes associated with differential binding (identified using ChIP-seq) for both drugs, showed that the top-downregulated molecular signatures were related to E response and MYC-targets in elacestrant versus fulvestrant-treated samples in both MCF7-LTED cell lines (Supplementary Fig. S4A, B).

RIME analysis also revealed similar effects of elacestrant and fulvestrant on the ER- interactome (Fig. 4C and Supplementary File S2), with only 3% of the ER interacting proteins differing between fulvestrant and elacestrant. To further explore the ER-interactome, we classified the identified proteins based on GO terms, such as hormone-binding proteins (GO:0051427) (Fig. 4D), transcription co-activator (GO:0003713) and co-repressor activity (GO:0003714) (Supplementary Fig. 5). Fulvestrant and elacestrant showed similar effects in most of the interactors as expected. However, among the hormone-binding proteins, only a small branch (highlighted in yellow) showed weaker ER binding with fulvestrant treatment in relation to elacestrant; this included proteins such as SIRT1, NCOR1 and SMARCA4 (Fig. 4D).

Taken together, these data suggest there is no clear evidence of distinct gene regulation changes related to elacestrant or fulvestrant and the drugs had very similar effects.

### Elacestrant impedes tumour progression in several human BC PDX models of endocrine resistance harbouring *ESR1* mutations

**Fig. 5:**
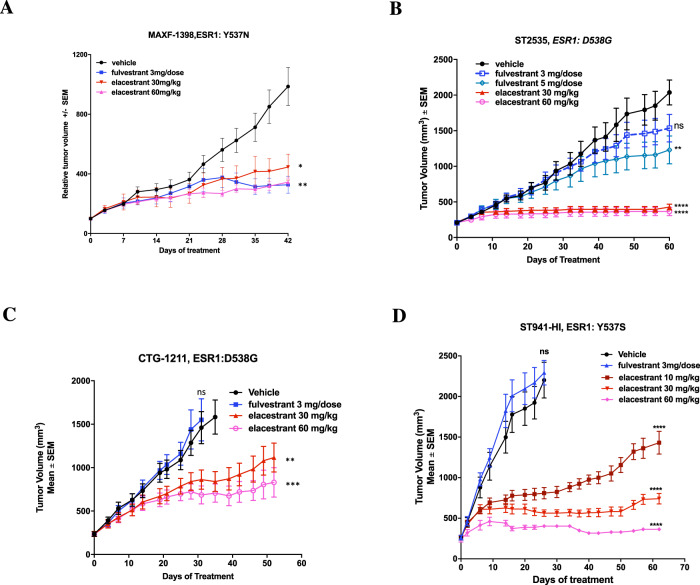
Effect of elacestrant versus fulvestrant on tumour progression in endocrine-resistant PDX models. **A** Long-term study assessing changes in tumour volume over 42 days of treatment in MAXF-1398, an ER+ model harbouring an *ESR1*^Y537N^ mutation and previously exposed to radiation and tamoxifen therapy but sensitive to fulvestrant (*n* = 5–6 animals/arm). **B**, **C** Long-term study assessing changes in tumour volume of treatment in **B** ST2535-HI (*n* = 7–10 animals/arm) and **C** CTG-1211-HI (*n* = 9 animals/arm) PDX models that are ER+ harbouring *ESR1*^D538G^ mutations and previously exposed to tamoxifen, AI and fulvestrant therapy. **D** Long-term study assessing changes in tumour volume over 64 days of treatment in ST941-HI, an ER+ model harbouring an *ESR1*^Y537S^ mutation and previously exposed to fulvestrant (*n* = 7–10 animals/arm). Mice were treated with vehicle control, fulvestrant (3 mg/dose) or elacestrant (10 mg/kg, 30 mg/kg or 60 mg/kg), as indicated. Data represent mean relative tumour volume (mm^3^) ± SEM. ANOVA with a Dunnett’s post-test was carried out for group comparisons. *p* values *p < 0.05, **p < 0.01, ***p < 0.001, ****p < 0.0001.

In order to assess the impact of elacestrant in vivo, we used several PDX models of acquired endocrine-resistant ER + BC. MAXF-1398^20^ harbours an *ESR1*^Y537N^ mutation and was derived from a patient previously exposed to radiation and tamoxifen therapy. The PDX is sensitive to fulvestrant, making it a good model to compare the efficacy of both SERDs (Fig. 5A). To address fulvestrant’s poor oral bioavailability, we used concentrations that were 2–3-fold higher than clinically achievable^16^. At the end of the course of treatment, both fulvestrant and two different concentrations of elacestrant showed significant reductions in tumour volumes (Fulvestrant: 64% reduction, *p* = 0.014; elacestrant (30 mg/kg): 55% reduction, *p* = 0.032; elacestrant (60 mg/kg): 67% reduction, *p* = 0.004) compared to vehicle control.

To further assess the effect of fulvestrant and elacestrant, we used two PDX models, ST2535-HI and CTG-1211-HI^21^, derived from patients previously exposed to tamoxifen, AI and fulvestrant therapy (Fig. 5B, C); both models harbour an *ESR1*^D538G^. Over the course of treatment, elacestrant showed a reduction of tumour volume for both PDX models compared to vehicle control. This effect was very similar irrespective of the dosage of elacestrant (ST2535-HI: 79% reduction, *p* < 0.0001 (30 mg/kg) and 82% reduction, *p* < 0.0001 (60 mg/kg); CTG-1211-HI: 30% reduction, *p* = 0.001 (30 mg/kg) and 48% reduction, *p* < 0.0001 (60 mg/kg)) compared to vehicle control. As expected, single agent fulvestrant showed little or no significant effect on tumour progression compared to vehicle control.

Similar effects were observed in a different model of fulvestrant resistance harbouring an *ESR1*^Y537S^ (ST941-HI) derived from a patient treated with an AI^21^ (Fig. 5D). Once again, fulvestrant showed no effect on tumour volume, whereas all three concentrations of elacestrant led to highly significant reductions in tumour volume in a dose-dependent manner (*p* < 0.0001) compared to vehicle control.

### Effects of elacestrant in combination with CDK4/6, PI3K and mTORC1 inhibitors

**Fig. 6:**
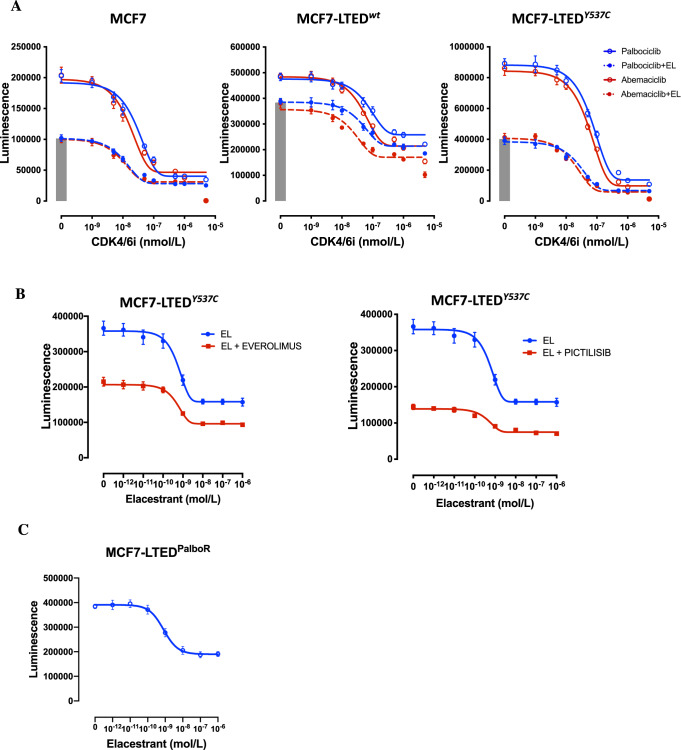
Effect of elacestrant in combination with targeted therapies. **A** Effect of escalating doses of palbociclib or abemaciclib with or without elacestrant in MCF7 (0.5 nM), MCF7-LTED^wt^ (20 nM) and MCF7-LTED^Y537C^ (1.5 nM) indicated by the bar in each graph. **B** Effect of escalating doses of elacestrant, with or without everolimus (5 nM) or pictilisib (100 nM) in MCF7-LTED^Y537C^. **C** Effect of escalating doses of elacestrant in MCF7-LTED^PalboR^. Data represented are mean ± SEM (*n* = 3 biological experiments with six technical replicates).

Recently, CDK4/6 inhibitors have shown marked efficacy in combination with aromatase inhibitors and fulvestrant in ER + BC. Based on this, we assessed the efficacy of combining elacestrant with the CDK4/6 inhibitors palbociclib and abemaciclib, as well as the mTORC1 inhibitor, everolimus, and PI3K inhibitor, pictilisib. In all MCF7 models, escalating concentrations of abemaciclib and palbociclib caused a dose-dependent decrease in proliferation with IC_50_ values of c.100 and 150 nM, respectively, in both MCF7 and MCF7-LTED^Y537C^. MCF7-LTED^wt^ showed slightly greater sensitivity to abemaciclib compared to palbociclib (IC_50_ 300 and 500 nM, respectively). In MCF7 and MCF7-LTED^Y537C^ cells addition of elacestrant appeared to act synergistically when combined with either abemaciclib or palbociclib, shifting the IC_50_ 2–3-fold. In the MCF7-LTED^wt^ cells, the addition of elacestrant caused a 30% drop in viability but increased the sensitivity of the cell line to abemaciclib, reducing the IC_50_ to c.100 nM (Fig. 6A).

As MCF7 and MCF7-LTED^Y537C^ have broadly similar sensitivity profiles to the drugs tested, we carried out experiments to assess the effect of mTORC1 suppression with everolimus on sensitivity to elacestrant in the MCF7-LTED^Y537C^ model (Fig. 6B). The IC_50_ concentration of everolimus alone caused suppression in viability. Everolimus in combination with elacestrant further reduced cell viability and showed additive activity, reducing the surviving fraction by a further 1.5-fold compared to elacestrant alone in the concentration range 1–1000 nM. Furthermore, pictilisib, showed a similar additive profile (Fig. 6B). Taken together, these data suggest that elacestrant in combination with either PI3K or mTORC1 blockade may provide effective therapy in endocrine-resistant BC.

### Effectiveness of elacestrant after resistance to palbociclib

The combination of CDK4/6 inhibitors and endocrine therapy has been shown to improve clinical outcomes in advanced ER + BC patients, however many patients will eventually relapse with acquired resistance. One major concern raised by clinicians relates to the in vitro analysis of CDK4/6 inhibitor-resistant cell lines showing reduced sensitivity to further endocrine blockade^22^. To explore this hypothesis, we subjected a palbociclib-resistant cell line generated from the MCF7-LTED^Y537C^ cells to escalating concentrations of elacestrant. Suppression of ER-signalling resulted in a dose-dependent decrease in cell viability with an IC_50_ of 10 nM compared to 5 nM in the parental cell line (Fig. 6C). This data suggests that elacestrant remains a viable treatment option in the palbociclib-resistant setting within the clinically achievable range.

### Generation and characterisation of elacestrant-resistant cell lines

**Fig. 7:**
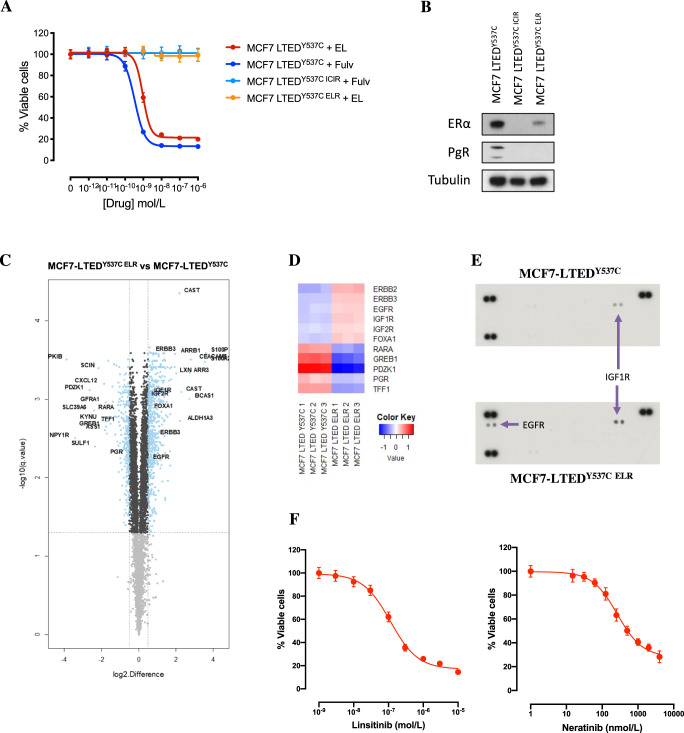
Mechanisms of resistance to elacestrant. **A** Effect of escalating concentrations of fulvestrant and elacestrant on the proliferation of MCF7-LTED^Y537C^ and its corresponding derivatives fulvestrant (ICIR) and elacestrant (ELR) resistant cell lines. Data represents % viable cells compared to vehicle control for each cell line. Error bars represent mean ± SEM (*n* = 3 biological experiments with six technical replicates). **B** Immunoblotting showing ER and PGR abundance (data representative of *n* = 3 biological replicates). **C** Volcano plot showing significant up- and downregulated proteins (blue) in MCF7-LTED^Y537C ELR^ cells in relation to the parental cell line (data generated from *n* = 3 biological replicates). Top significant proteins, E-regulated targets and growth factors are highlighted. **D** Heatmap representation of the protein expression of E-regulated targets, ER modulators and growth factors in MCF7-LTED^Y537C^ parental versus MCF7-LTED^Y537C ELR^. **E** Reverse phase protein arrays assessing receptor tyrosine kinase expression. **F** Effect of escalating concentrations of linsitinib and neratinib on the proliferation of MCF7-LTED^Y537C ELR^ cell lines. Data represents % viable cells compared to vehicle control for each cell line. Error bars represent mean ± SEM.

To address the potential resistance mechanisms associated with long-term treatment with fulvestrant and elacestrant, MCF7-LTED^Y537C^ cells were cultured long-term in the presence of elacestrant (1000 nM) or fulvestrant (100 nM) until they developed resistance (Fig. 7A).

Elacestrant-resistant derivative (MCF7-LTED^Y537C ELR^) showed a reduction in the abundance of ER in relation to the parental MCF7-LTED^Y537C^ but with levels slightly higher than the fulvestrant-resistant line (MCF7-LTED^Y537C ICIR^). In addition, PGR abundance was also reduced in both MCF7-LTED^Y537C ICIR^ and MCF7-LTED^Y537C ELR^ (Fig. 7B).

To further explore the mechanisms of resistance to elacestrant, we carried out a proteomic analysis, which revealed 1972 up- and 2110 downregulated proteins in MCF7-LTED^Y537C ELR^ compared to its parental cell line (FDR <5%; Supplementary File S3 and Fig. 7C). Although classic ERGs were mainly inhibited in MCF7-LTED^Y537C ELR^, we detected upregulation of the pioneer transcription factor FOXA1 that is able to modulate ER activity (Fig. 7D). Inhibition of FOXA1 by siRNA knockdown suppressed the proliferation of MCF7-LTED^Y537C ELR^ (Supplementary Fig. 6). Furthermore, MCF7-LTED^Y537C ELR^ showed activation of ERBB2/EGFR, MAPK and AKT oncogenic signatures (Fig. 7D). In addition, we used reverse phase protein arrays to assess changes in receptor tyrosine kinase abundance. EGFR and IGF-1R were both upregulated (Fig. 7E), confirming our proteomic analysis (Fig. 7D and Supplementary File S3).

We further investigated the role of IGF-1R and EGFR as drivers of resistance to elacestrant by assessing sensitivity to escalating concentrations of inhibitors, linsitinib and neratinib. A dose-dependent decrease in cell viability with an IC_50_ of 200 nM (linsitinib) and 500 nM (neratinib) was observed (Fig. 7F). Taken together, these data showed that loss of ER-signalling together with increased EGFR and IGF-1R activity acted as new mitogenic drivers.

## Discussion

To date, fulvestrant is the only SERD approved for clinical use, but due to its poor oral bioavailability and pharmacokinetics, some patients show sub-optimal occupancy of the ER, even at the highest doses achievable and this restricts clinical effectiveness^23^. This, coupled with the intramuscular route of administration, has led to the initiation of several research programs that seek to develop oral SERDs with improved pharmacology and bioavailability. To date, several oral SERDs and SERM/SERD hybrids have been reported^13,14,24,25^; the furthest in development is elacestrant^16,26^, which is currently in phase III clinical trial in patients with advanced or metastatic ER + breast cancer^17,27,28^.

In this report, we have expanded on previous studies of elacestrant^16^ to provide a comprehensive analysis of the effect of elacestrant versus fulvestrant on ER-signalling in vitro and in PDX models of endocrine-sensitive and -resistant ER + BC by assessing the effect of both drugs on the ER-transcriptome, proteome and cistrome. Elacestrant and fulvestrant reduced the proliferation of both endocrine-sensitive and -resistant models. The IC_50_ for elacestrant was, on average, tenfold higher than for fulvestrant, but it remained within the clinically achievable concentration range. One caveat to this observation is that in vitro models do not recapitulate fulvestrant’s poor oral bioavailability and this needs to be considered when comparing the clinical relevance of dose-related comparisons.

*ESR1* mutations are strongly associated with endocrine-resistant metastatic BC and in particular upon relapse on aromatase inhibitor therapy^3^. Studies of the most common mutations suggest they alter the conformation of helix 12, promoting the AF-2 domain to lie in an activated conformation in the absence of a ligand. Nonetheless, this conformation remains capable of ligand binding, which has led to the hypothesis that mutant-ER may remain sensitive to direct antagonism^8,29^. This concept is supported by recent retrospective clinical studies, which have shown that *ESR1* mutations are sensitive to fulvestrant^30^ but that much higher concentrations of the drug are required to target the mutated receptor and, in particular, ESR1^Y537S^ and ESR1^D538G^^9–12^. To test if the partial resistance conferred by ER mutations was a class effect for SERDs, we compared the effects of elacestrant and fulvestrant using in vitro models of resistance to LTED, which harbour naturally occurring *ESR1* hotspot mutations (Y537C and Y537S). Although elacestrant targeted the *ESR1* mutations, higher concentrations of the drug were required compared to the parental cell lines harbouring *ESR1*^*wt*^ and the antiproliferative effect was comparable to that of fulvestrant.

To address the clinical implications of this observation, we examined the effects of both drugs in vivo in PDX models with varying *ESR1* mutations. MAXF-1398, which harbours a *ESR1*^*Y537N*^ mutation, was equally sensitive to both fulvestrant and elacestrant. It is noteworthy that this mutation, similar to ESR1^Y537C^, is less prevalent^31^ and may bind ligands more effectively. In contrast, PDX models harbouring ESR1^D538G^ and ESR1^Y537S^, which were resistant to fulvestrant (ST2535-HI, CTG-1211-HI, ST941-HI), showed sensitivity to elacestrant. This observation was in contrast to both our in vitro model of fulvestrant resistance and also our *ESR1*^*Y537S*^ mutant cell line models. There are several potential explanations for these observations. Firstly, in our cell line model of acquired fulvestrant resistance ER expression is lost, while clinically, most patients who relapse on fulvestrant show reduced, but not entirely suppressed ER levels^32^. Consequently, due to the poorer pharmacokinetics of fulvestrant, residual ER remains un-targeted and capable of driving proliferation. Secondly, studies show that ESR1^Y537S^ and ESR1^D538G^ alter the structure of helix 12 significantly, thereby reducing its affinity for ligands^33^ and as such, much higher concentrations of fulvestrant are required to impact ER degradation, an observation which has been associated with elevated toxicity^10^. In contrast, elacestrant which shows improved bioavailability and pharmacokinetic properties, is capable of targeting both residual wild-type and mutated *ESR1*^16,34^, a finding supported clinically in the recent phase I trial of elacestrant where anti-tumour activity was observed in heavily pre-treated patients with metastatic BC including patients whose tumours harboured *ESR1* mutations as well as in patients who had progressed on fulvestrant^27^.

Comparative assessment of clinically achievable concentrations of elacestrant and fulvestrant on the ER-transcriptome showed that elacestrant downregulated the expression of several key ER-regulated genes (*TFF1*, *GREB1*, *PGR*, *PDZK1* and *TFF3*)^35^ to a greater extent than fulvestrant. While overall, both drugs caused very similar changes in the ER-cistrome and consistent changes in global gene expression, some subtle differences were evident. For instance, elacestrant appeared to target pathways involved in proliferation more strongly, while fulvestrant showed a higher effect on inflammatory-associated pathways. Consistent with the overall similarity, limited changes in the ER-interactome were evident. Taken together, these data suggest elacestrant, like fulvestrant, acts as a pure anti-oestrogen.

Assessments of changes in the global protein profiles from cell line models treated with fulvestrant or elacestrant showed higher levels of residual ER in elacestrant-treated versus fulvestrant-treated cells and a concordant lower level of E3-ligases. These data are in keeping with our InCell assay, which showed that fulvestrant was a superior ER-degrader compared to elacestrant. One explanation for this observation relates to the molecular structure of fulvestrant. Fulvestrant is a 7α-alkylsulphinyl analogue of 17β-estradiol^36^ and has a chemical structure which is distinct compared to nonsteroidal SERMs such as tamoxifen and raloxifene and the third-generation SERM bazedoxifene^25^. Fulvestrant-bound-ER prevents receptor dimerisation, and energy-dependent nucleo-cytoplasmic shuttling, thereby blocking nuclear localisation of the receptor^37,38^. Furthermore, any nuclear fulvestrant-bound-ER is transcriptionally inactive as both AF1 and AF2 are disabled^39–41^. Finally, fulvestrant has a long hydrophobic tail which, when the drug is bound to the ligand binding domain of ER, results in destabilisation of the receptor and subsequent degradation via the ubiquitin-E3-ligase pathway. Elacestrant, while showing improved inhibitory potency as a result of its pharmacokinetics and anti-oestrogenic activity, does not destabilise the ER to the same degree in vitro. One explanation may be that elacestrant’s chemical structure is closer to that of a SERM and that upon binding, it does not disrupt helix 12 to the same degree as fulvestrant; its pure anti-oestrogenic pharmacology appears to be more dependent on the competitive antagonism of ER. Indeed, recent x-ray crystallographic studies have shown that elacestrant adopts a novel conformation in the ERα ligand binding pocket and forms a unique hydrogen bond that is not observed in other competitive anti-oestrogens and uses a chemical space known to increase helix 12 mobility and induce SERD activity^42^. This novel conformation also places it near positions 537 and 538, the two most common sites of somatic mutations in ER.

Studies have shown that *ESR1*-mutant breast cancers may harbour other alterations, including elevated PI3K/AKT pathway activation, amplification of cyclin D1, together with increased growth factor signalling via FGF and ERBB receptors^31,43^. We, therefore, assessed the efficacy of elacestrant in combination with inhibitors targeting CDK4/6 and PI3K/AKT/mTOR; as expected, combinations appeared superior to monotherapy.

Lastly, we investigated potential resistance mechanisms associated with long-term elacestrant exposure. Unlike fulvestrant-resistant cell lines, elacestrant-resistant lines continued to express measurable levels of ER, although they were significantly reduced.

Proteomic profiling showed increased levels of FOXA1 (which, when suppressed, reduced cell proliferation) and increased growth factor signalling together with dependence on EGFR and IGF-1R, evidenced by sensitivity to inhibitors targeting these receptors.

FOXA1 is a pioneer factor and major determinant of ER-driven transcriptional activity^44^ and its expression has been associated with good response to endocrine therapy in luminal A breast cancers^45^. However, aberrant expression of FOXA1 has also been implicated in resistance to endocrine therapy. In this context, FOXA1 has been shown to alter the ER-cistrome leading to the expression of genes which potentiate more aggressive behaviour such as IL-8, which promotes tumour cell survival and metastasis^46^. Moreover, recent studies have linked FOXA1 in this context to the regulation of super-enhancers which leads to genome-wide enhancer re-programming^47^ resulting in the activation of prometastatic genes controlling epithelial-mesenchymal transition^48^. This high degree of enhancer re-programming results in tumour plasticity^47,48^. Additionally, direct links between FOXA1 and IGFR signalling have been shown. For instance, in lung cancer, FOXA1 increases expression of IGF-IR^49^, whilst in luminal B breast cancer, FOXA1 has been shown to regulate insulin growth factor 1 (IGF1) activity. In this context, IGF-I increases the stability of FOXA1 protein, which in turn influences the expression of genes modulated by IGF-I and consequently IGF-I-mediated biological responses^50^. These findings emphasise the importance of understanding the role of FOXA1 further and exploring the potential of using it as a therapeutic target in ER-positive breast tumours. It is, therefore clear these pathways should eventually be considered worthy of investigation as candidate-acquired resistance mechanisms to elacestrant in the clinic.

In conclusion, our molecular and cytologic studies indicate that elacestrant, like fulvestrant, is a pure anti-oestrogen. Elacestrant’s improved oral bioavailability and pharmacokinetics, and its capacity to target mutant-ER as well as fulvestrant resistance support its continued investigation as a potential alternative to fulvestrant in the clinic. Additionally, it may be well-suited as a partner with other drugs that target non-ER-based endocrine resistance mechanisms.

## Materials and methods

### Reagents and antibodies

Reagents were obtained from the following sources: 17-β-estradiol (E2), 4-hydroxytamoxifen and 4-androstenedione (Sigma-Aldrich); Fulvestrant (Tocris); Linsitinib (MedChemExpress) everolimus, pictilisib, palbociclib and abemaciclib (SelleckChem). Elacestrant was supplied by Radius Health Inc.

### Cell culture

Human breast cancer cell lines MCF7, T47D, HCC1428 and SUM44 were obtained from the American Type Culture Collection, USA and Asterand and were cultured in phenol red-free RPMI1640 medium supplemented with 10% foetal bovine serum and 1 nM E2. All cell lines were banked in multiple aliquots to reduce the risk of phenotypic drift and identity was confirmed using STR. Cells were routinely screened for mycoplasma contamination. Long-term E-deprived (LTED) cells modelling resistance to an AI were derived from all parental cell lines as previously described^18,51^. Two models of MCF7-LTED cells were available: one harbouring a Y537C mutation in *ESR1* and the other with wt-*ESR1*. SUM44-LTED was shown to harbour a natural heterozygous *ESR1*^Y537S^ mutation.

ICIR and TAMR cell lines were cultured in their respective basal medium supplemented with 100 nM fulvestrant (ICI182780) or 100 nM 4-hydroxytamoxifen. CRISPR-Cas9 engineered MCF7-LTED^∆537C^ cells were generated, as previously described^12^. Elacestrant-resistant cell lines were generated by growing parental cells (MCF7-LTED^Y537C^) long-term in the presence of RPMI1640 containing 10% DCC + 0.1 nM E2 + 1 μM elacestrant. All cell lines were stripped of steroids for 48–72 h prior to the start of experiments. Ishikawa cells were grown in RPMI1640 medium containing 10% DCC-FBS.

### Proliferation assays

Cells were stripped of E2 by culture in RMPI1640 containing 10% DCC for 48 h prior to seeding into 96-well tissue culture plates. Monolayers were allowed to acclimatise for 24 h prior to treatment with RPMI1640 + 10% DCC containing increasing concentrations of drugs. The medium was replaced after 3 days, and cells were cultured for a total of 5–6 days. Cell viability was determined using the CellTitre-Glo® Luminescent Cell Viability Assay (Promega), according to the manufacturer’s protocol. Values were expressed as % viability relative to the vehicle-treated control. Spheroid cultures were generated by seeding 2500 cells per well of a 96-well ultra-low attachment plate (Costar). Plates were spun at 900×*g* for 10 min. Spheres were formed over 72 h and subsequently treated with the drugs as indicated for 7 days. Proliferation was assessed using Celigo S (Nexcelom Bioscience).

### SiRNA knockdown

MCF7 cells were reverse-transfected. siRNA upon receipt were prepared as 20 mM stocks and stored at −20 °C (ON-TARGETplus Human FOXA1 siRNA [Entrez 3169]; ON-TARGETplus Non-targeting Control siRNAs from Horizon). For 24-well plates, the siRNA was diluted to a concentration of 2 mM using serum-free RPMI1640 and 6 ml was added to 994 ml of serum-free RPMI1640 to give 12 pmol per well of a 24-well plate. Lipofectamine RNAiMAX (Thermofisher) 1 ml was added to each well. Plates were gently mixed and incubated for 10–20 min at room temperature. Cells were diluted in RPMI1640 to a final concentration of 60,000 cells/ml. About 500 ml of cells was added to each well, giving a final number of 30,000 per well of a 24-well plate. The following day the medium was changed to the relevant selection and incubated for 72 h. Monolayers were then washed and subjected to immunoblotting. For 96-well plates, each well was treated with 2.4 pmol per well for each siRNA and 0.2 ml of Lipofectamine RNAiMAX in 20 ml of RPMI1640. Cells were diluted to 15,000/ml and 100 ml added to each well to give a final concentration of 1500/well. Plates were incubated overnight and then the medium was changed to the appropriate selection. Plates were incubated for a further 5 days and proliferation was assessed using CellTitre-Glo® Luminescent Cell Viability Assay (Promega).

### Immunoblotting

Whole-cell extracts were generated as described previously^52^. In brief, cell monolayers were washed with ice-cold phosphate-buffered saline and then lysed in extraction buffer (1% (v/v) Triton X-100, 10 mm Tris-HCl, pH 7.4, 5 mm EDTA, 50 mm NaCl, 50 mm sodium fluoride, 2 mm Na_3_VO_4_, containing Complete™ inhibitor mix (Roche Applied Science) per 10 ml of buffer) and homogenised by passage through a 26-gauge needle six times. The lysate was incubated on ice for 10 min prior to centrifugation (14,000 rpm at 4 °C). Equal amounts of protein were resolved by SDS-PAGE using 4–12% gradient gel (Biorad) and then subjected to immunoblot analysis. Antigen-antibody interactions were detected with ECL-reagent (Amersham, UK). Proteins were detected using the following antibodies: ER (ERα(F-10) sc-8002, Santa Cruz Biotechnology) at 1:200, cyclin D1 (CST-2922, Cell Signalling Technology) at 1:1000, PGR (NCL-L-PGR, Novocastra) at 1:500, FOXA1 (Abcam; ab 5089) at 1:1000, GAPDH [6C5] (Abcam; ab 8245) at 1:2000 and Tubulin (T-9026, Sigma- Aldrich) at 1:5000. Uncropped immunoblots are show in Supplemental Fig. 7.

### RNA-seq

Libraries were created after Ribo-zero rRNA removal kit (Illumina) using NEBNext Ultra Directional RNA (NEB) and sequenced using the HiSeq2500 (paired-end 100 bp v4 chemistry). Tophat (v2.1) and DESeq2 (V2.2)^53^ with default parameters were used for alignment and differential expression analysis. Gene set enrichment analysis (GSEA)^54^ was used to identify gene sets that were significantly up/downregulated in each treatment. Data were deposited in GSE190384 and GSE190386 (http://ncbi.nlm.nih.gov/geo/).

### Chromatin immunoprecipitation (ChIP)

ChIP-seq was performed, as previously described^55,56^, using ER antibody (HC-20 (sc-543) Santa Cruz) 10 μg per pull-down. Only binding events that occurred in two biological replicates were considered differential binding sites using Diffbind v1.14.5^57^ and R v3.2.1. Data GSE190385 is deposited at http://ncbi.nlm.nih.gov/geo/.

### Phospho-receptor tyrosine kinase arrays

Analysis of phosphoproteins was carried out using the Proteome Profiler Human Phospho- RTK array kit (R&D systems), according to the manufacturer’s instructions. Briefly, cells were treated with drugs for 24 h, lysed and incubated with membranes spotted with capture antibodies. Following incubation with HRP-labelled secondary antibodies, membranes were exposed to radiography film.

### InCell western blot analysis

Cells (2 × 10^4^/well) were plated in clear-bottom 96-well black plates for 24 h prior to the addition of ligand for 18 h. Cells were fixed using 3.7% paraformaldehyde and permeabilised with PBS containing 0.1% Triton X-100. Detection of ERα was carried out using the Novocastra 6F11 antibody (Leica) at 1:200. IRDye 800CW goat anti-mouse conjugate was used to detect binding. Analysis was performed as per LICOR manufacturer’s protocol using the LICOR ODYSSEY CLx imaging system. Data were normalised using CellTag 700 stain (LICOR).

### Alkaline phosphatase assay

Cells were grown for 72 h in RPMI1640 medium containing 10% DCC-FBS and were then seeded into 96-well plates for a further 24 h. The medium was then replaced with medium containing either control or treatment and the plates were incubated at 37 ^o^C. After 1 and 6 days, the cells were washed with PBS and then lysed by freezing at −80 °C for 30 min. The alkaline phosphatase activity was determined using a 1-Step PNPP Kit (Pierce) following the manufacturer’s instructions. Briefly, after thawing the frozen cells, 1-Step PNPP solution was added to each well and mixed with the cell extracts. The plates were then incubated at room temperature (RT) for 1 h on an orbital shaker. Stop solution (2 N NaOH) was added to each well, mixed and the lysates were transferred into a fresh 96-well plate for colorimetric assessment. The absorbance was measured at 405 nm using a Victor X5 plate reader (PerkinElmer, UK).

### qPLEX-RIME

ER-interactome was evaluated using qPLEX-RIME, as previously described, using ER (Abcam, ab-3575) and IgG (Santa Cruz Biotechnology, sc-2027)^58^. The tryptic digests were isobarically labelled with tandem-mass tags (TMT) (10-plex-labelled, Pierce 90110), mixed and subjected to high pH fractionation prior to LC-MS/MS. The raw data were analysed using MaxQuant v1.6.0.16. The corrected reporter ion intensities were log2 transformed and median normalised per channel at the protein level. All proteomics data were deposited within the PRIDE database (PXD031377).

### Quantitative analysis of global protein expression

Cells were lysed and labelled using the iFASP (isobaric mass tagging with Filter-Aided Sample Preparation) protocol^59^. In brief, cells were lysed in 8 M UREA and 50 mM TEAB. About 100 μg for each condition were individually reduced using TCEP, alkylated using chloroacetamide and digested using trypsin in FASP. The tryptic digests were isobarically labelled with tandem-mass tags (TMT) (10-plex-labelled, Pierce 90110), mixed and subjected to high pH fractionation prior to LC-MS/MS. The raw data were analysed using MaxQuant v1.6.0.16. The corrected reporter ion intensities were log2 transformed and median normalised per channel at the protein level. All proteomics data were deposited within the PRIDE database (PXD031377).

### In vivo patient-derived xenografts

All study protocols were reviewed by Radius, approved by Institutional Animal Care and Use Committees (IACUC) and conducted in accordance with the US and International regulations for the protection of laboratory animals. Female athymic nude mice (NU(NCr)-Foxn1nu or BALB/cAnNCrl-Foxn1nu) were obtained from Envigo RMS, Inc., Jackson laboratories, Harlan Laboratories, or Charles River Laboratories and acclimated for 3 to 7 days prior to implantation. The ST2535-HI (hormone-independent) and the ST941-HI PDX models were derived and studied at South Texas Accelerated Research Therapeutics (San Antonio, TX). The CTG-1211-HI PDX model was derived and studied at Champions Oncology (Rockville, MD). The MAXF-1398 PDX model was derived at studied at Charles River Discovery, Oncotest GmbH (Germany). All animals were subcutaneously implanted with PDX models. When tumours grew to 150–200 mm^3^, mice were randomised based on tumour volume and administered the indicated treatments. Tumours were measured twice weekly with Vernier calipers; volumes were calculated using the formula: (L × W^2^) × 0.5, where L = length and W = width in mm of the tumour. Elacestrant (Radius, Inc) was administered orally and daily for the duration of study. Preformulated, clinical-grade fulvestrant (Faslodex, AstraZeneca) was obtained through third-party vendors and administered by subcutaneous injection once weekly. At the end of study, tumours were harvested 4 h post-last dose unless otherwise indicated. Tumour growth inhibition (%TGI) was calculated as [1−(average relative tumour volume treatment group/average relative tumour volume vehicle group)] × 100.

### Statistical analysis and data analysis

Statistical and graphical presentations were performed using GraphPad Prism 7. For cell proliferation assays, the IC_50_ was calculated by fitting a dose-response curve using a nonlinear regression model with a log(inhibitor) versus response curve fit. For all xenograft studies, tumour volumes were represented as mean ± standard error of the mean (SEM). Statistical evaluations of the differences between groups were assessed using one-way ANOVA with Dunnett’s post-test. Statistical significance was defined as n.s. *P* ≥ 0.05, *P ≤ 0.05, **P ≤ 0.001, ***P ≤ 0.001, ****P ≤ 0.0001. Figure legends show the tests used for each part of the study. Quantitative data were presented as the mean ± SEM, unless stated otherwise.

## Supplementary information

Supplemental Material

Supplementary File 1

Supplementary File 2

Supplementary File 3

## Data Availability

The datasets supporting the conclusions of the current study are publicly available upon reasonable request. Proteomic data is held on the PRIDE database (PXD031377). Whilst RNA-seq and ChIP-seq (GSE190386) are held within the NCBI GEO repository http://ncbi.nlm.nih.gov/geo/ ChIP-seq data (GSE190385) and RNA-seq GSE190384.
