# Supplementary material for: Elacestrant demonstrates strong anti-estrogenic activity in PDX models of estrogen-receptor positive endocrine-resistant and fulvestrant-resistant breast cancer
Source: NPJ Breast Cancer. 2022 Nov 29;8:125. doi: 10.1038/s41523-022-00483-1 (PMC9709100; doi:10.1038/s41523-022-00483-1)
Supplement: Supplementary file 1 — Supplemental Material [file 41523_2022_483_MOESM1_ESM.pdf]

Supplementary Table 1 Genotype of study cell lines showing *ESR1*, *PIK3CA* and *PTEN* mutation status

Cell line	ESR1	PIK3CA	PTEN
MCF7-LTED^{wt}	Wild-type	mutant	Wild-type
MCF7-LTED^{Y537C}	Y537C	mutant	Wild-type
MCF7-LTED$^{\Delta 537C}$	Wild-type	mutant	Wild-type
MCF7-TAMR	Wild-type	mutant	Wild-type
MCF7-LTED^{ICIR}	Y537C	mutant	Wild-type
MCF7-LTED^{ELR}	Y537C	mutant	Wild-type
MCF7-LTED^{PalboR}	Y537C	mutant	Wild-type
SUM44	Wild-type	Wild-type	Wild-type
SUM44-LTED^{Y537S}	Y537S	Wild-type	Wild-type
T47D	Wild-type	mutant	Wild-type
T47D-LTED	Wild-type	mutant	Wild-type
HCC1428	Wild-type	Wild-type	Wild-type
HCC1428-LTED	Wild-type	Wild-type	Wild-type

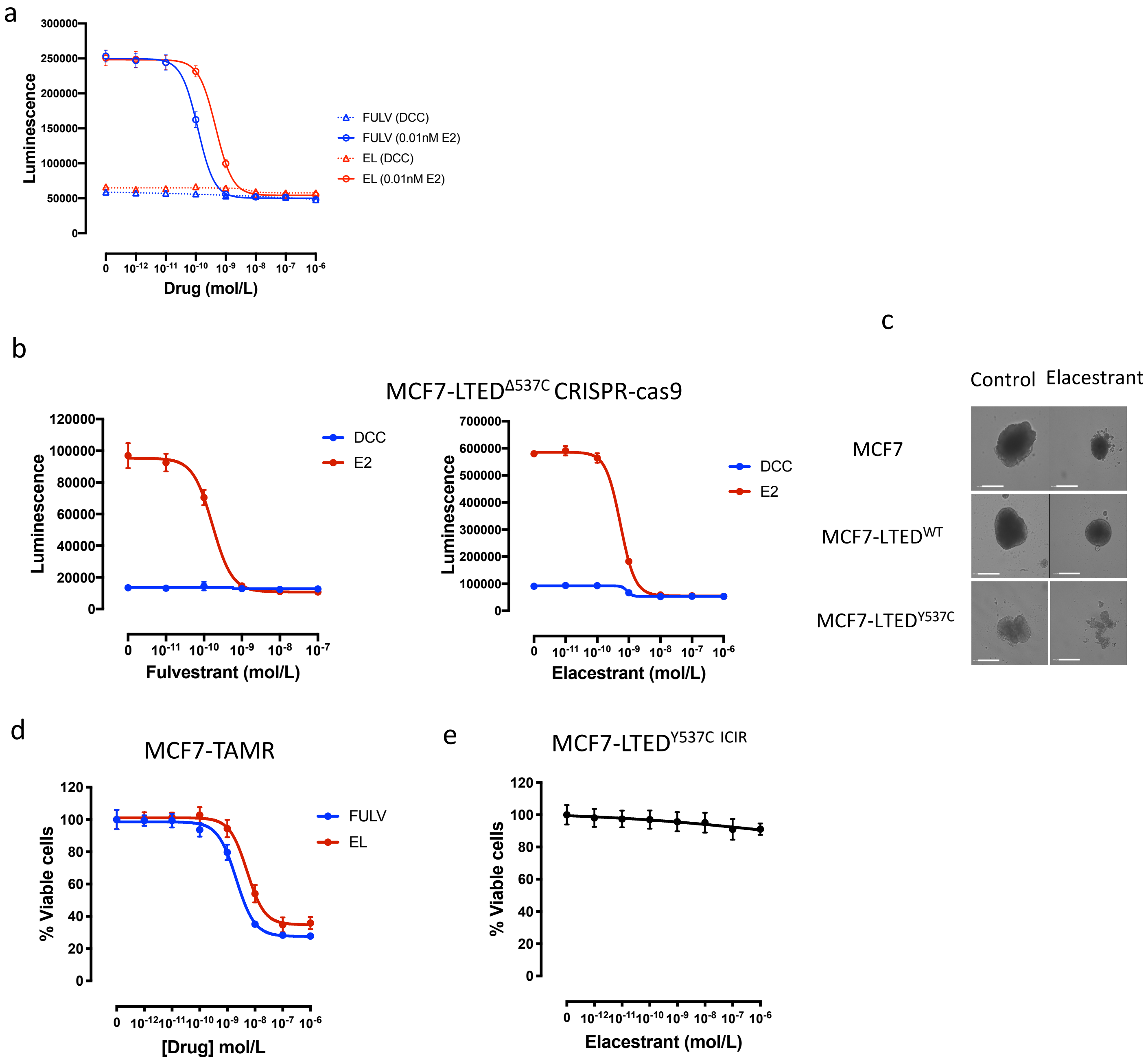

Supplementary Figure 1. (a) Effect of escalating doses of elacestrant (EL) or fulvestrant (FULV) in MCF7 cell lines. Proliferations were performed in the presence or absence of 0.01nM 17b-estradiol (E2). Cell viability was analysed using a CellTiter-Glo assay. Data are expressed as absolute luminescence. Error bars represent means ± SEM. Red and blue shaded bars represent the clinically achievable doses for elacestrant and fulvestrant, respectively. (b) Effect of escalating doses of elacestrant (EL) or fulvestrant (FULV) in models of MCF7-LTED$^{\Delta537C}$ CRISPR-Cas9 modified cell lines. Data are expressed as absolute luminescence. (c) Antiproliferative effect of elacestrant in 3D spheroid models of MCF7, MCF7-LTED^{wt} and MCF7-LTED^{Y537C}. (d) Effect of escalating concentrations of elacestrant (EL) or fulvestrant (FULV) in MCF7 model of tamoxifen resistance (TAMR); (e) Effect of escalating doses of elacestrant in MCF7 cells models with acquired sequential resistance to LTED (MCF7-LTED^{Y537C}) followed by fulvestrant (MCF7-LTED$^{Y537C\ ICIR}$).

Ishikawa endometrial cell lines

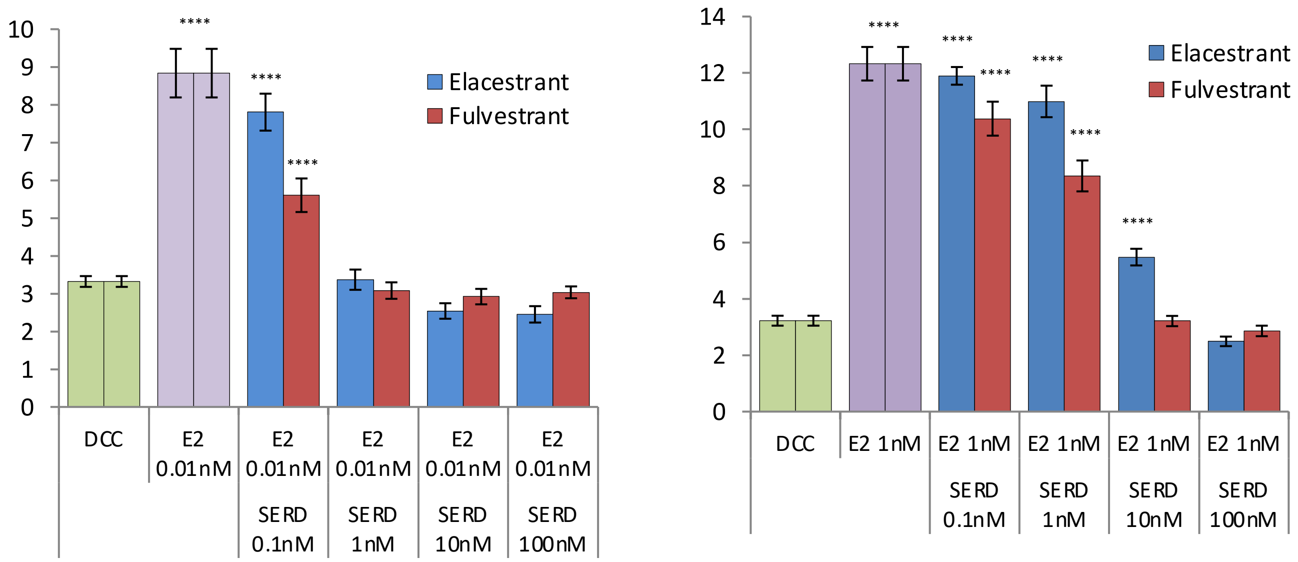

Supplementary Figure 2. Alkaline phosphatase assay for a competition analysis in the presence of either 0.01nM E2 (-11) or 1nM E2 (-9) and increase doses of fulvestrant or elacestrant in Ishikawa endometrial cell line. Bars represent the mean ± SEM of n=3 three independent experiments with n=6 technical replicates. **** $p<0.0001$, *** $p<0.001$ (one ANOVA, with Dunnett's multiple comparisons test)

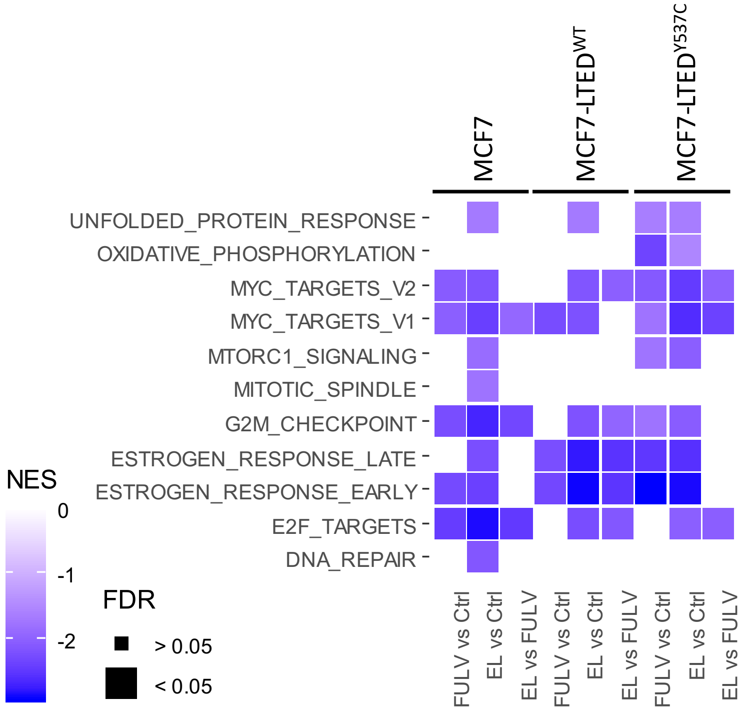

Supplementary Figure 3. Heatmap showing the inhibited pathways following treatment with 10nM fulvestrant (FULV) or 100nM elacestrant (EL) in wt-MCF7, MCF7-LTED^{wt} and MCF7-LTED^{Y537C} based on global protein expression analysis.

a

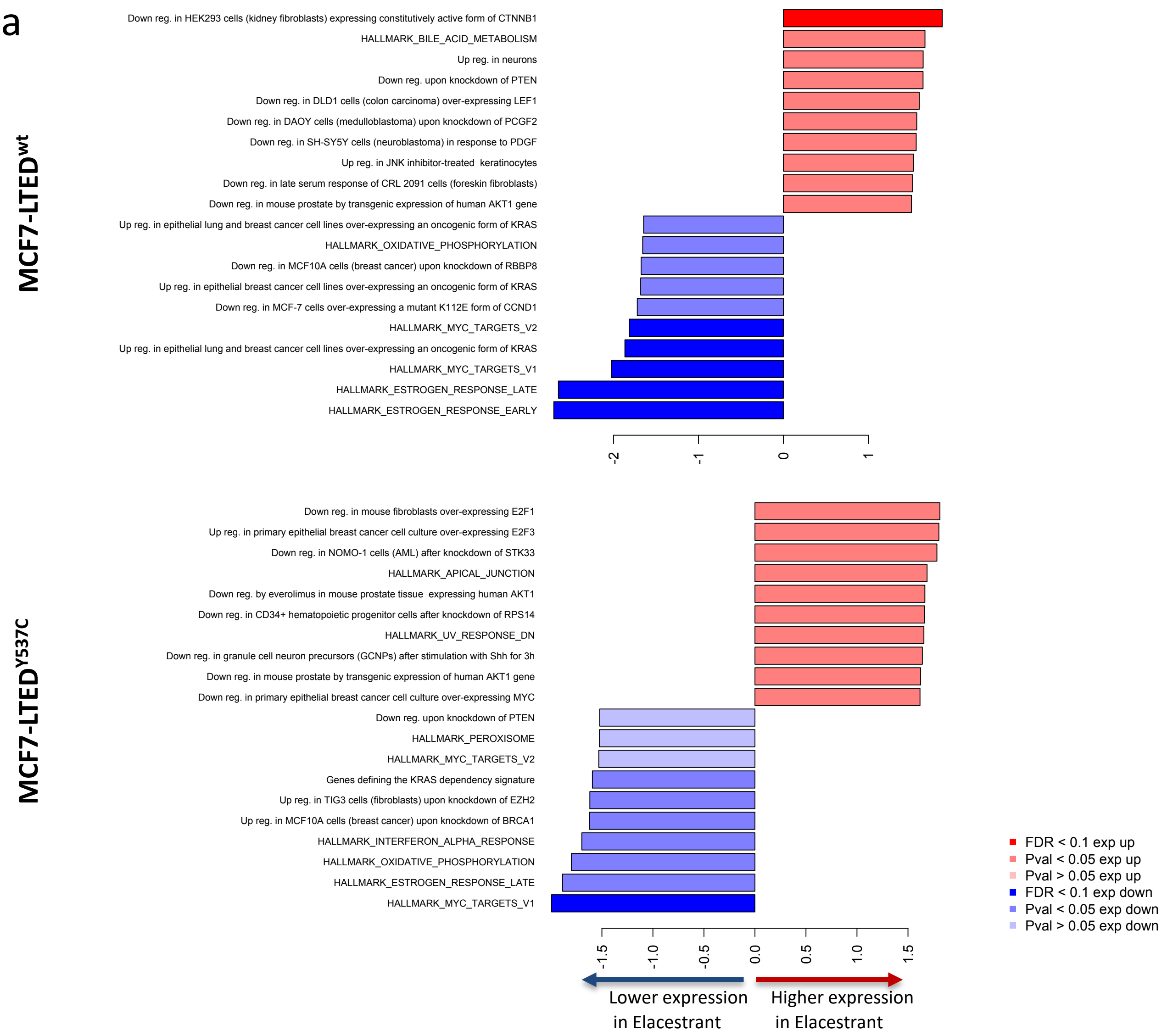

b

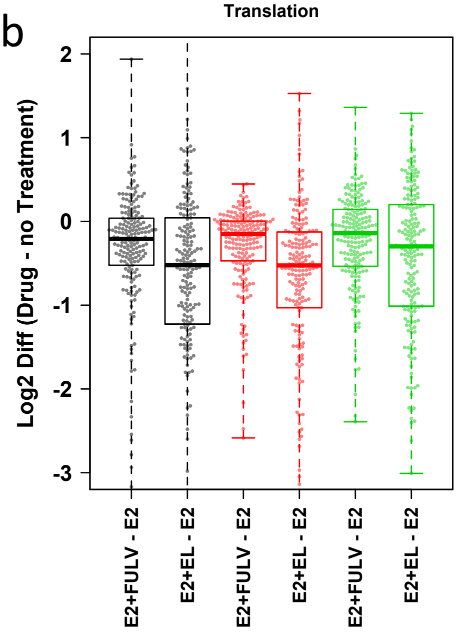

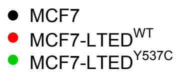

Supplementary Figure 4. (a) Molecular signatures identified by GSEA analysis of overlap between RNA-seq genes differentially expressed and ChIP-seq genes associated with differential binding between fulvestrant and elacestrant in MCF7-LTEDwt and MCF7-LTED^{Y537C} (FDR < 0.05). (b) Effect of elacestrant [(E2+EL) -E2] or fulvestrant [(E2+FULV) -E2] on the expression of GO translational genes.

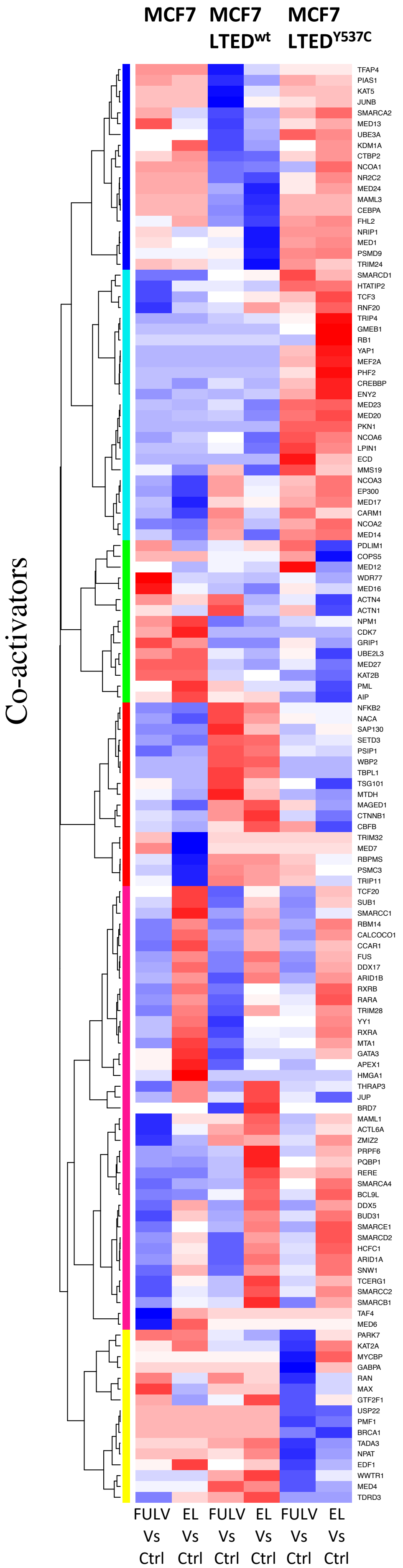

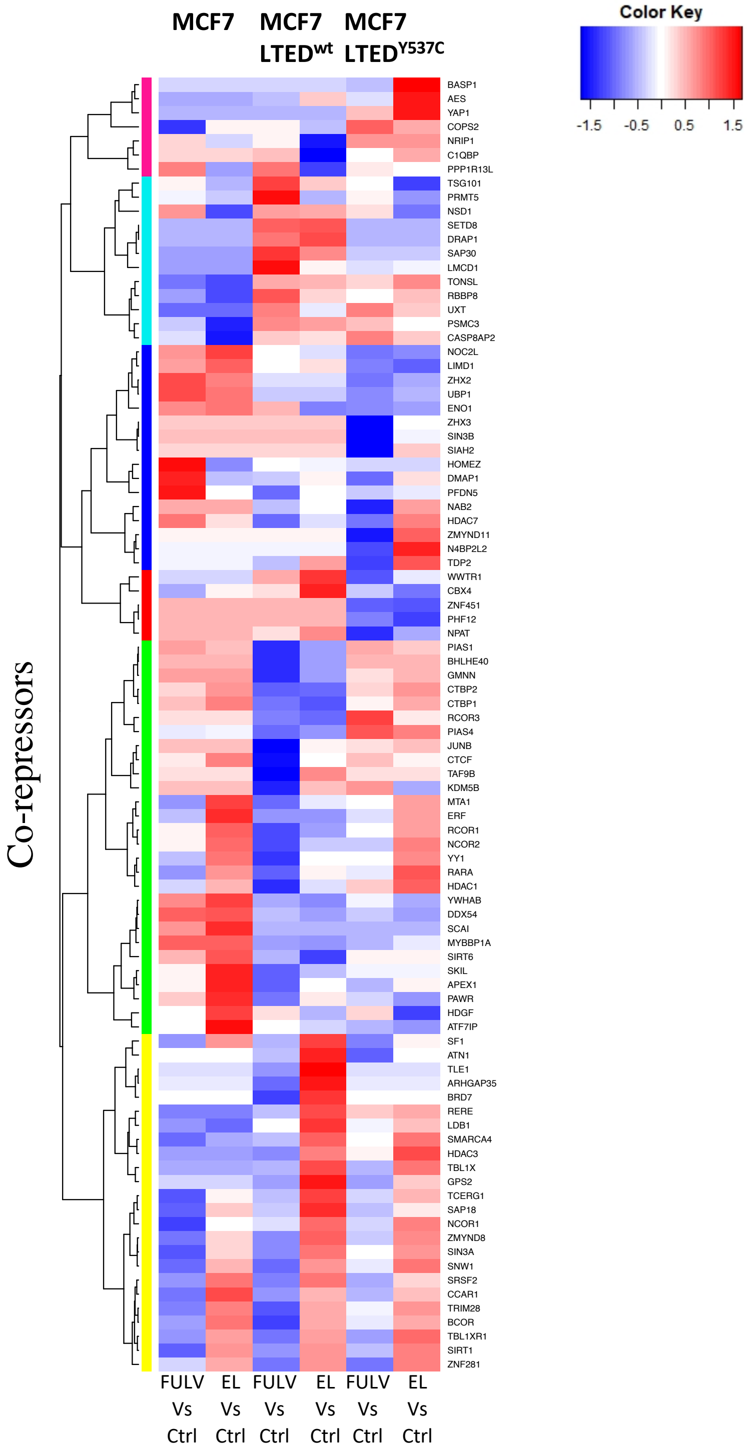

Supplementary Figure 5. Heatmap showing GO co-activator and co-repressor proteins that interacted in cells treated with fulvestrant (FULV) (10nM) or elacestrant (EL) (100nM) in relation to the control (Ctrl) (E2, 1nM). Fulvestrant and elacestrant showed similar effect in most of the interactors with exception the co-activators in the branch highlighted in pink and co-repressors in the branch highlighted in yellow. p-value based on Student's t test or Pearson correlation is shown; r: Pearson`s correlation score.

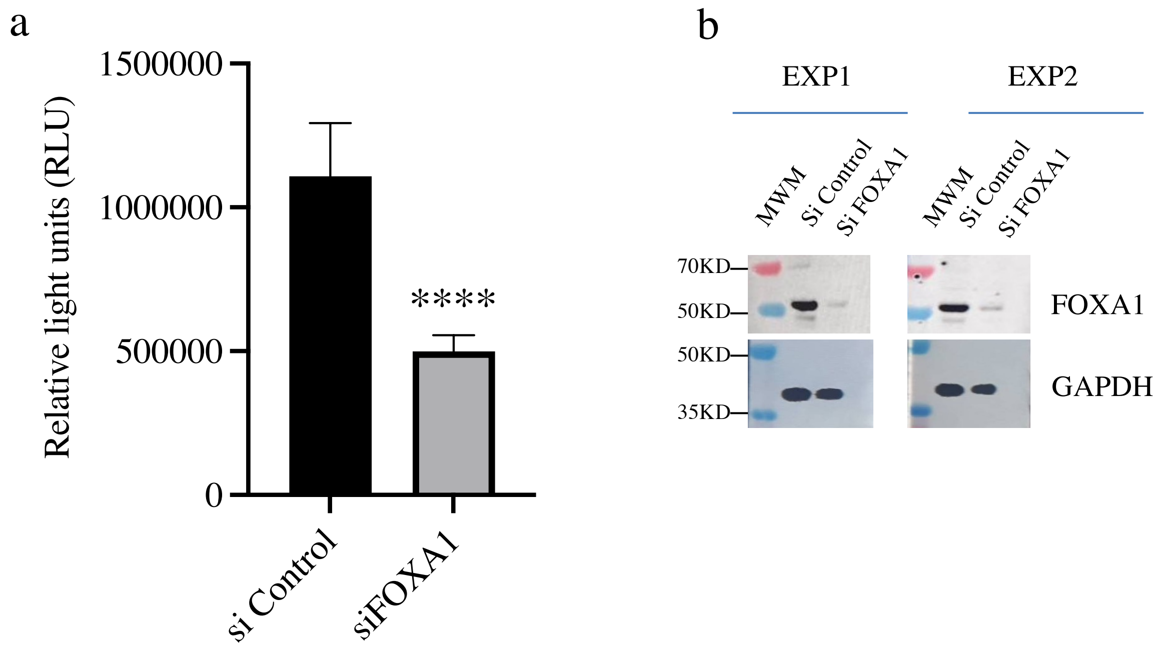

Supplementary Figure 6 (a) Impact of siFOXA1 on MCF7-LTED$^{Y537C\ ELR}$ compared to siControl. Cells were reverse transfected using Lipofectamine RNAiMAX (Thermofisher) according to the manufacturer's instructions with each siRNA in 96 well plates and treated for 5 days with medium containing elacestrant. Proliferation was assessed using Titreglo. Absolute relative light units are shown (n=3 biological experiments each with 8 technical replicates) ($p>0.0001$) p value is based on an unpaired t-test. (b) An accompanying 24 well plate was treated similarly and harvested after 72 hours to assess changes in FOXA1 abundance. GAPDH was used as a loading control.

Full western blot of PGR (blue box) used for figure 7b Lane 1, MCF7 LTED ^{Y537C}, 2. MCF7 LTED ^{Y537CICR} 3. MCF7 LTED $^{Y537C\ ELR}$ (2 minute exposure)

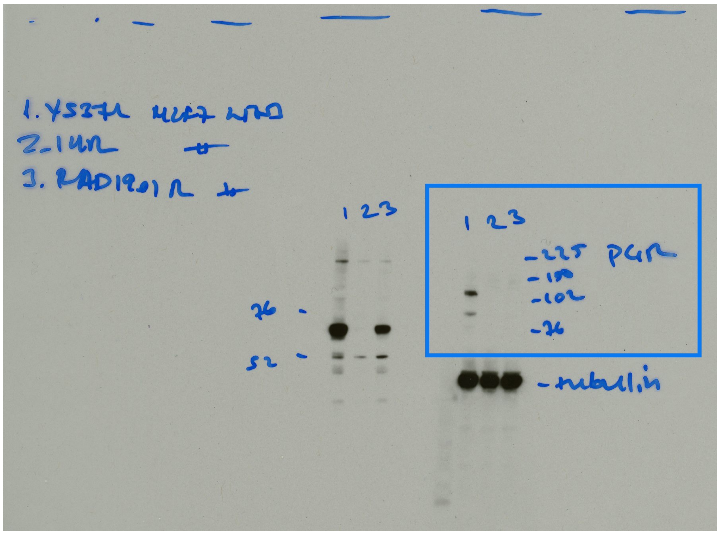

Full western blot of ER and tubulin (blue box) used for figure 7b Lane 1, MCF7 LTED ^{Y537C}, 2. MCF7 LTED ^{Y537CICR} 3. MCF7 LTED $^{Y537C\ ELR}$ Not ER and tubulin immunoblot is lower exposure of PgR immunoblot shown above hence PGR is not visible (2 sec exposure)

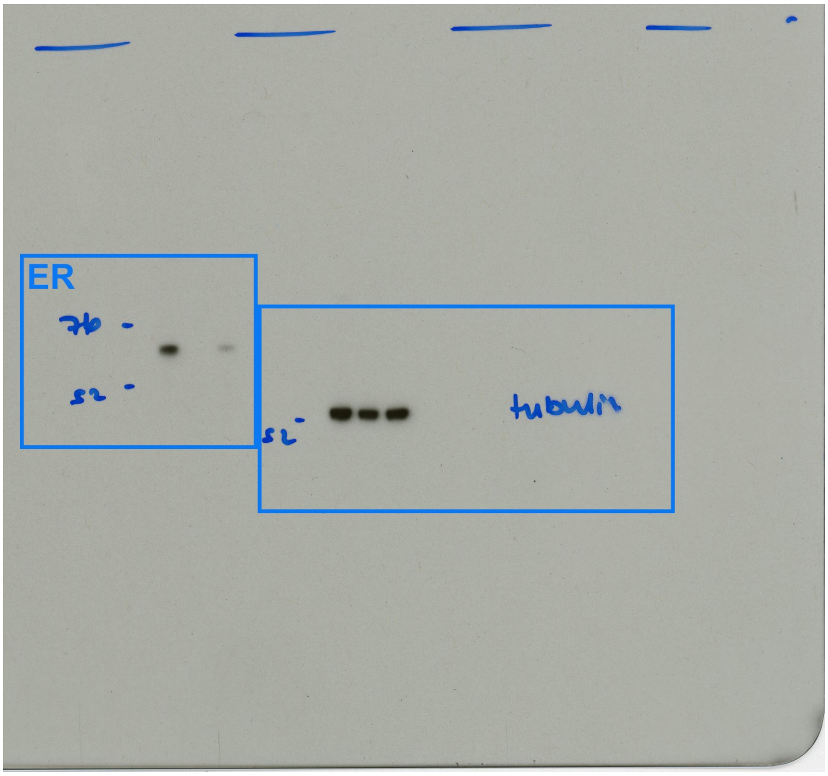

Supplemental Figure 7 Shows the uncropped immunoblots used in the study and the figures they relate to.

Full western blots for figure 2b **Wt- MCF7.** White boxes show the data for A. ER, B. CCND1 C. PGR D. Tubulin. Note blots a-c are different exposure to capture alterations.

a. ER b. CCND1 c. PGR d. Tubulin

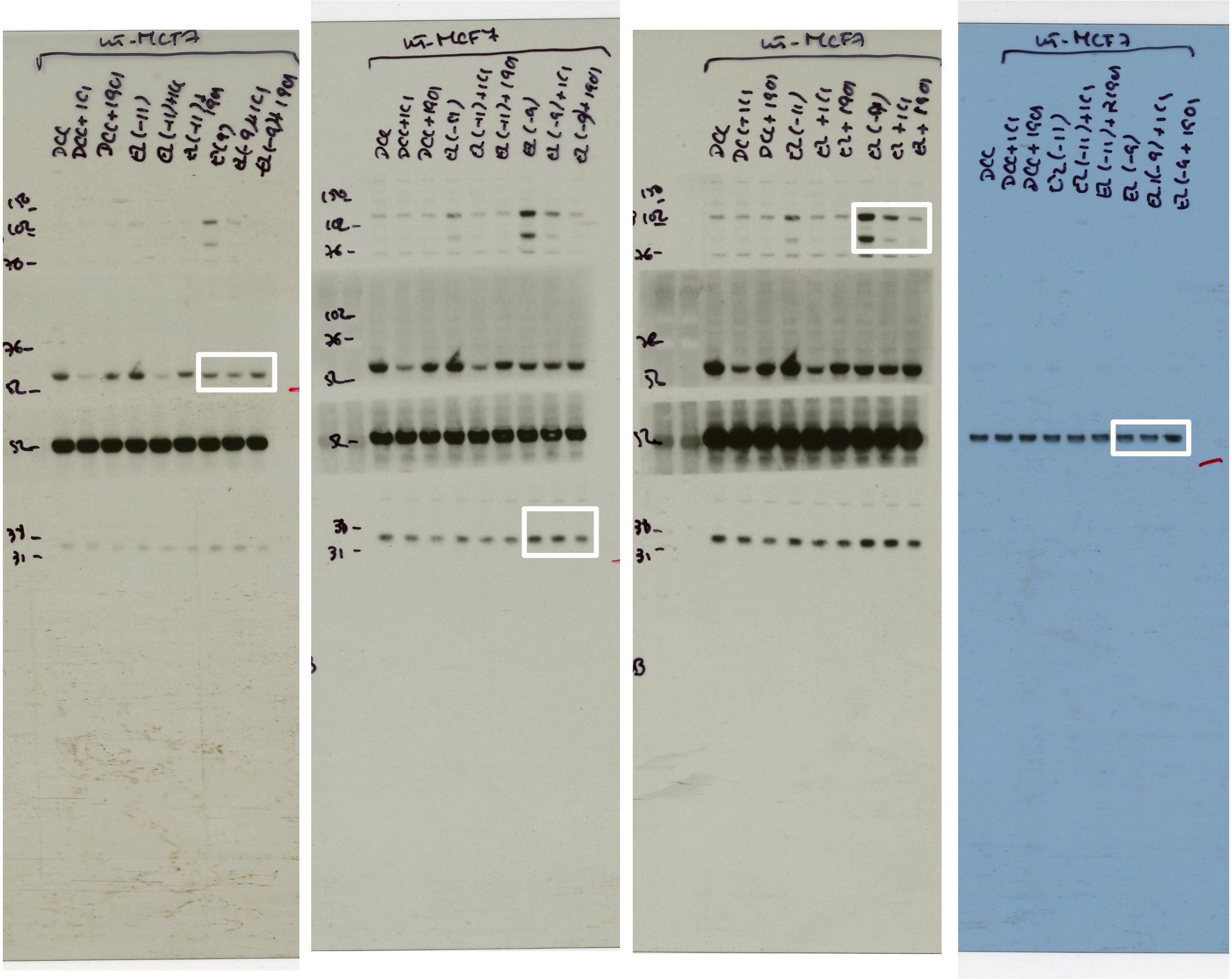

Supplemental Figure 7 Shows the uncropped immunoblots used in the study. Continued

Full western blots for figure 2b **MCF7 LTED** Wt. White boxes show the data for a. ER, b. CCND1 and Tubulin and c. PGR not this blot is a longer exposure of blot b in order to detect PGR

a. ER

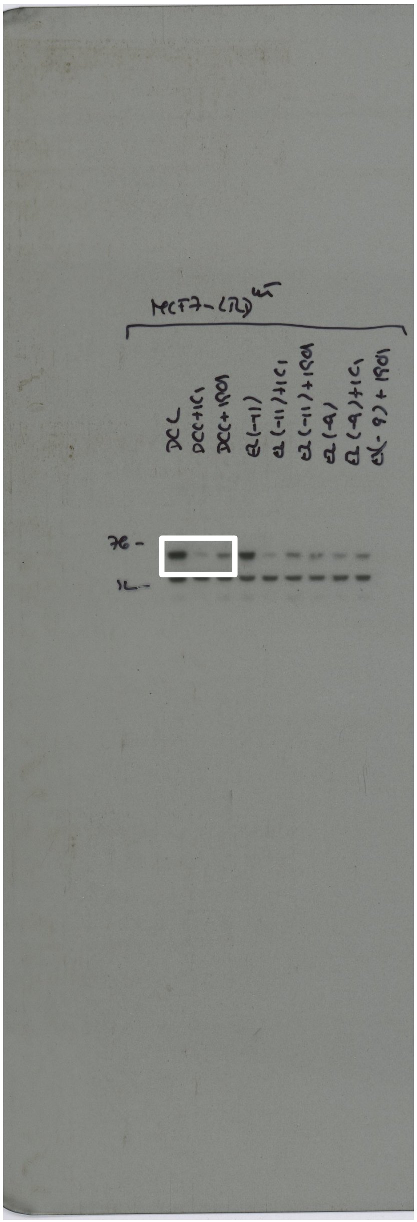

b. CCND1 and Tubulin

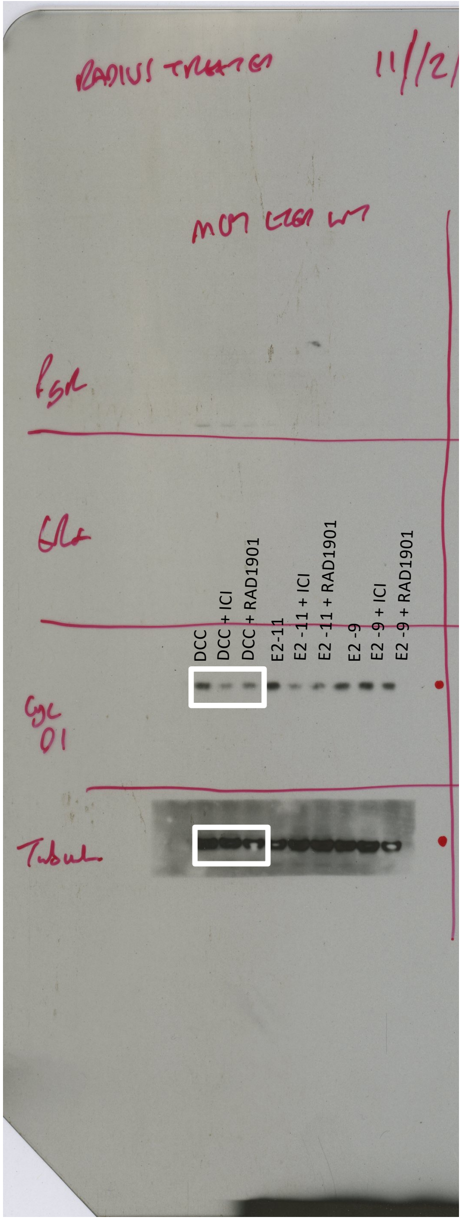

c. PGR

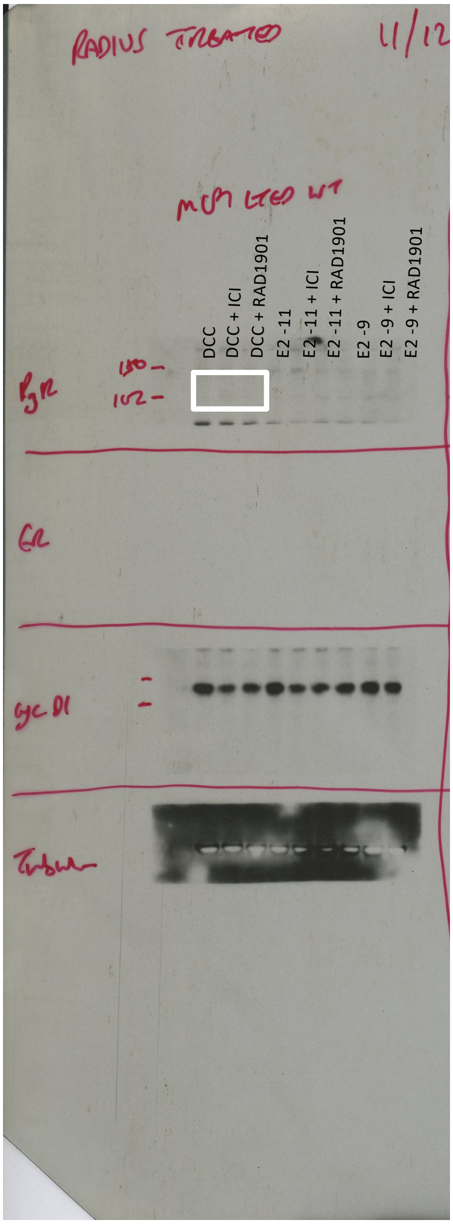

Supplemental Figure 7 Shows the uncropped immunoblots used in the study. Continued

Full western blots for figure 2B **MCF7 LTED** ^{Y537C}. White boxes show the data for a. ER and tubulin, b. PGR and CCND1

a. ER and Tubulin

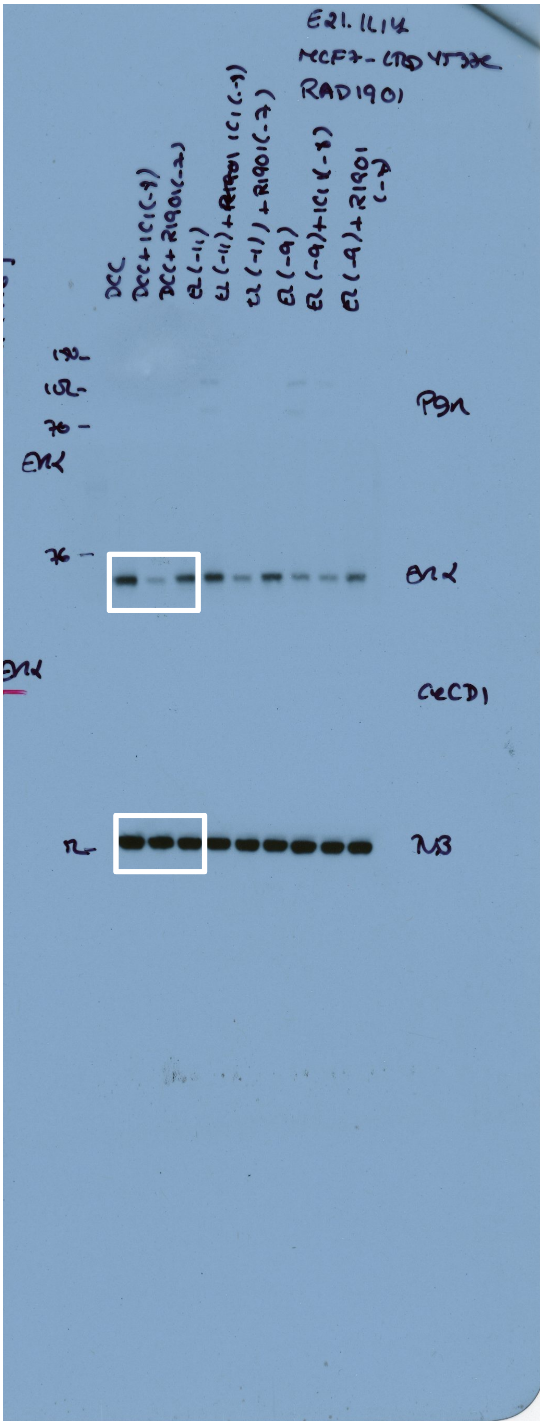

b. PGR and CCND1

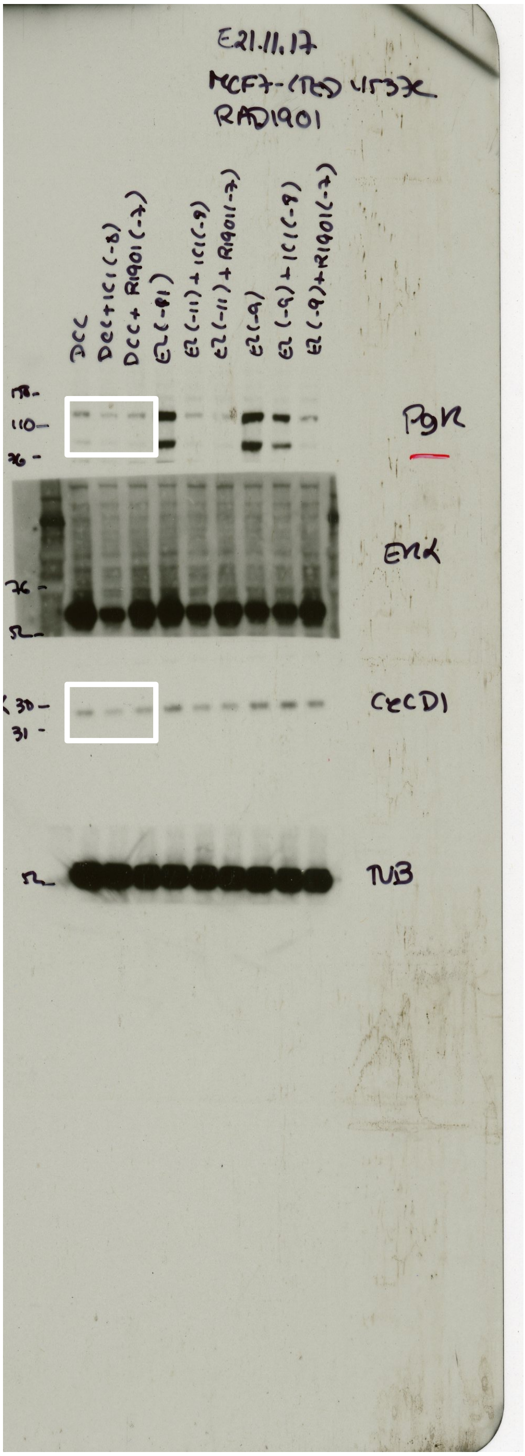

Supplemental Figure 7 Shows the uncropped immunoblots used in the study. Continued

Full uncropped immunoblot for supplemental Figure 6

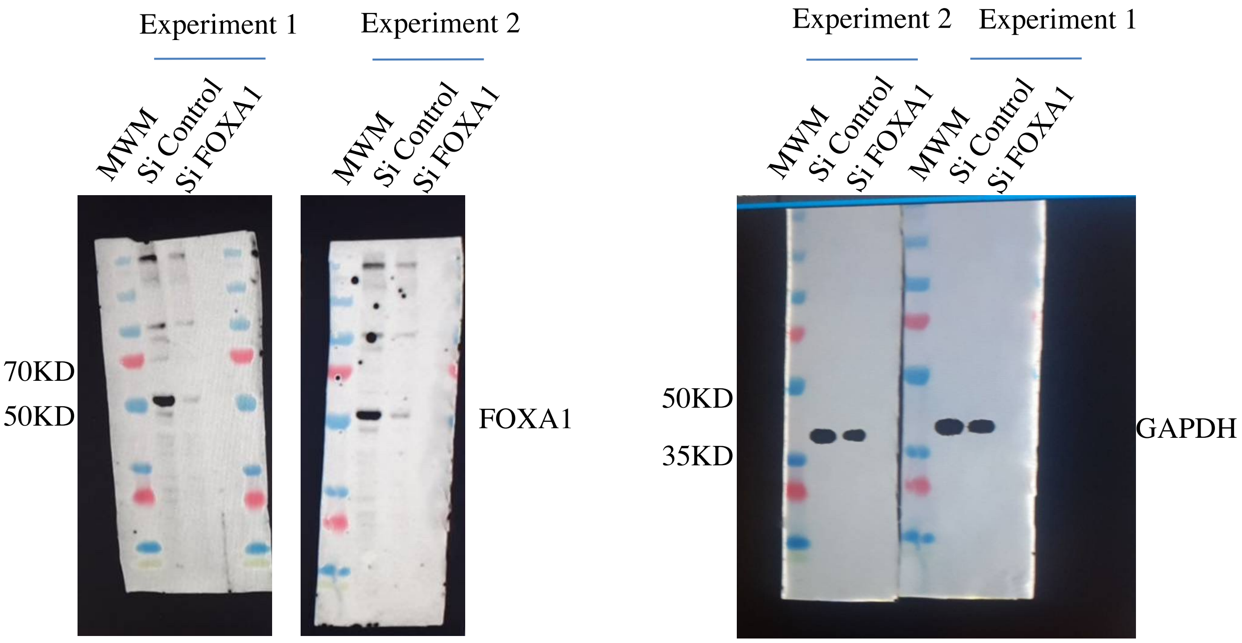

Supplemental Figure 7 Shows the uncropped immunoblots used in the study. Continued
